# Data-driven analyses of motor impairments in animal models of neurological disorders

**DOI:** 10.1371/journal.pbio.3000516

**Published:** 2019-11-21

**Authors:** Hardeep Ryait, Edgar Bermudez-Contreras, Matthew Harvey, Jamshid Faraji, Behroo Mirza Agha, Andrea Gomez-Palacio Schjetnan, Aaron Gruber, Jon Doan, Majid Mohajerani, Gerlinde A. S. Metz, Ian Q. Whishaw, Artur Luczak

**Affiliations:** 1 Canadian Center for Behavioural Neuroscience, University of Lethbridge, Lethbridge, Alberta, Canada; 2 Coastline Automation, San Jose, California, United States of America; 3 Faculty of Nursing & Midwifery, Golestan University of Medical Sciences, Gorgan, Iran; University of Minnesota, UNITED STATES

## Abstract

Behavior provides important insights into neuronal processes. For example, analysis of reaching movements can give a reliable indication of the degree of impairment in neurological disorders such as stroke, Parkinson disease, or Huntington disease. The analysis of such movement abnormalities is notoriously difficult and requires a trained evaluator. Here, we show that a deep neural network is able to score behavioral impairments with expert accuracy in rodent models of stroke. The same network was also trained to successfully score movements in a variety of other behavioral tasks. The neural network also uncovered novel movement alterations related to stroke, which had higher predictive power of stroke volume than the movement components defined by human experts. Moreover, when the regression network was trained only on categorical information (control = 0; stroke = 1), it generated predictions with intermediate values between 0 and 1 that matched the human expert scores of stroke severity. The network thus offers a new data-driven approach to automatically derive ratings of motor impairments. Altogether, this network can provide a reliable neurological assessment and can assist the design of behavioral indices to diagnose and monitor neurological disorders.

## Abstract 2

This study demonstrates that a state-of-the-art neural network can provide automated scoring of motor deficits with an accuracy equivalent to that of human experts and has the potential teach us to develop more-sensitive behavioral tests.

## Introduction

Classification and quantification of behavior is central to understanding normal brain function and changes associated with neurological conditions [1,2]. Investigations of neurological disorders are aided by preclinical animal analogues that include laboratory rodents such as rats and mice. Whereas hand use is important to most human activities, rodents also use their hands for building nests, digging, walking, running, climbing, pulling strings, grooming, caring for young, and for feeding—essentially, for much of their behavior. A number of laboratory tests have been developed to assess skilled hand use in rodents, including having an animal reach into a tube or through a window to retrieve a food pellet or having an animal operate a manipulandum or pull on a string to obtain food [3–11]. In addition, skilled walking tasks assess rodent fore- and hind limb placement on a narrow beam or while crossing a horizontal ladder with regularly or irregularly spaced rungs [12–17]. Most of the tests for rodents have been developed as analogues that assess human neurological disorders. For example, a test of skilled reaching for a single food item is used as a motor assessment of rodents and nonhuman primates as well as for the human neurological conditions of stroke [18,19], Parkinson disease [20–22], and Huntington disease [23].

There are many ways of assessing forelimb reaching movements, including end-point measures that give a score for success or failure, kinematic procedures that trace the Cartesian trajectory of a limb segment, and notational scores that describe the relative contributions of different body segments to a movement. Here, scoring of animal and human reaching was done based on the Eshkol-Wachman movement notational system, which treats the body as a number of segments. Each movement is scored in terms of those body segments that contribute to the movement [24,25]. For example, a normal act of reaching for food by a rat or human can be divided into several movement elements: hand lifting, hand advancing, pronating, grasping, etc. [9,22,26]. If a brain injury impairs the movement of the limb, a subject may still successfully reach; however, the features of reaching may significantly differ from a normal reach. For instance, the angle of the hand during advancing to reach a food item may significantly differ in stroke versus control animals. The notational scoring system captures and quantifies these changes [3,19,27,28].

Primary disadvantages of using descriptive notational analysis are that a scorer needs to acquire expertise with the system, the procedure is time-intensive and so limits the analysis to sampling, and scoring is subject to human bias and so usually requires more than one scorer to obtain interrater reliability. A solution to these problems is the development of automated methods for movement analyses that can replace or complement manual scoring. Recent advancements in deep neural networks have achieved impressive accuracy in many image recognition tasks (e.g., [29–32]) and offer a promising approach for automated behavioral analyses [33–35].

Here, we demonstrate that deep neural networks can provide fully automatic scoring for fine motoric behaviors, such as skilled reaching, with human expert accuracy. The neural network presented here was also successful in scoring other behavioral tasks. The main contribution of the present study is to demonstrate a method for extracting knowledge from deep neural networks in order to identify movement elements that are most informative for distinguishing normal and impaired movement. This procedure offers a data-driven method for discovering the most-predictive movement components of neurological deficits, which, in turn, can guide development of more-sensitive behavioral tests for the detection and monitoring of neurological disorders.

## Results

### Design of a deep neural network to automatize and to achieve reproducibility of behavioral analyses

**Fig 1:**
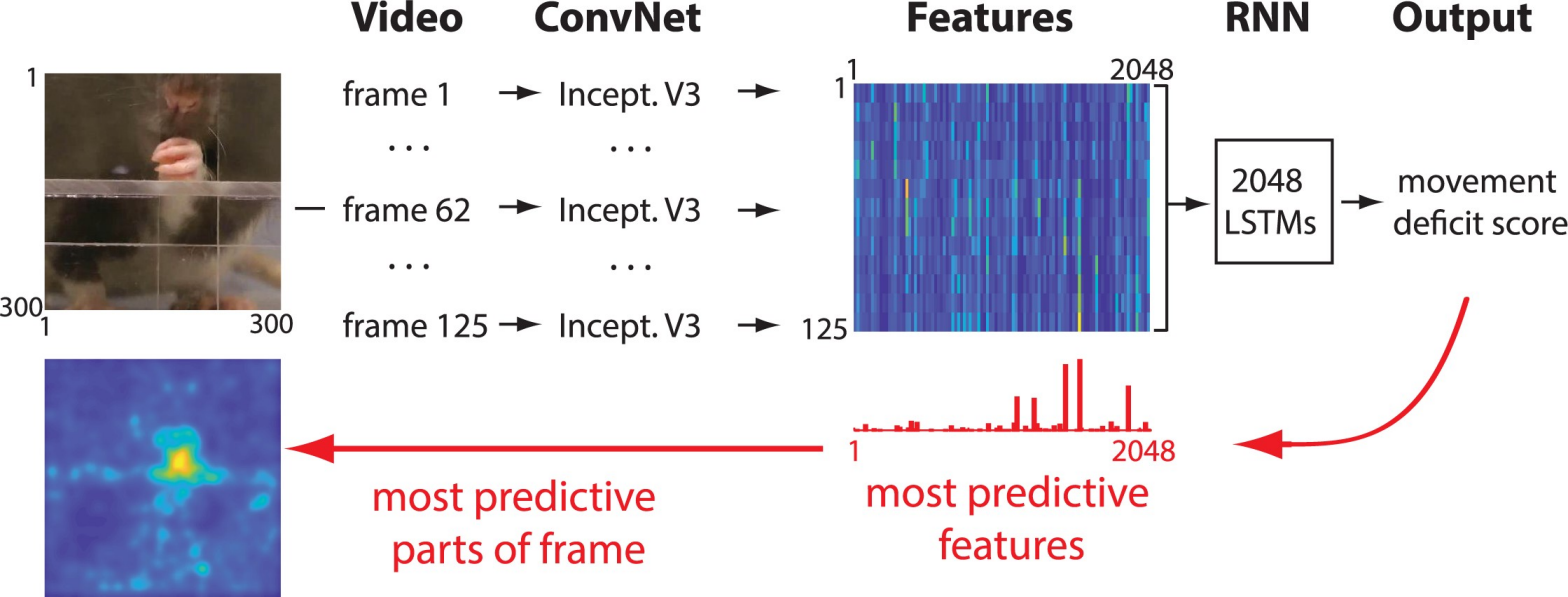
Network architecture. Each frame is first passed through a ConvNet, called Inception V3 (“Incept. V3”), that reduces dimensionality by extracting high-level image features [36]. The features from 125 successive video frames are then given as an input to an RNN. The RNN is composed of LSTM units with the capacity to analyze temporal information across frames. The RNN outputs the movement deficit score for each video. After the network is trained, information is extracted from the network weights in order to identify image features and the parts of each video frame that were most predictive of the network score (red arrows). Network code is available at github.com/hardeepsryait/behaviour_net, and weights of trained model are available at http://people.uleth.ca/~luczak/BehavNet/g04-features.hdf5. See Methods for details. ConvNet, convolutional network; LSTM, long short-term memory; RNN, recurrent neural network.

Our network was composed of two parts. The first consisted of a convolutional network (ConvNet), Inception-V3 [36] (Methods). The function of the ConvNet was to convert each video frame (300 × 300 pixels) to a set of 2,048 features to reduce the dimensionality of the data. By analogy, this could be thought of as transforming an image from the retina into neuronal representations in higher-order visual areas that represent complex features of the original image [37]. Next, the features from 125 video frames from a single video clip (sampled at 30 frames/second) were combined and passed to a recurrent neural network (RNN) that analyzed the temporal information in the movements of an animal or human participant (Fig 1). The network was then trained to assign a movement deficit score for each video clip that matched the score from a human expert (Methods). After the network was trained, we applied recently developed methods for knowledge extraction [38,39] (Methods) to identify which movement features were most informative to the network in discriminating control from stroke animals. With the same methodology, the parts of each video frame that were most informative for the network decision were identified (Fig 1).

### Comparison of movement deficits scores between expert and the network

**Fig 2:**
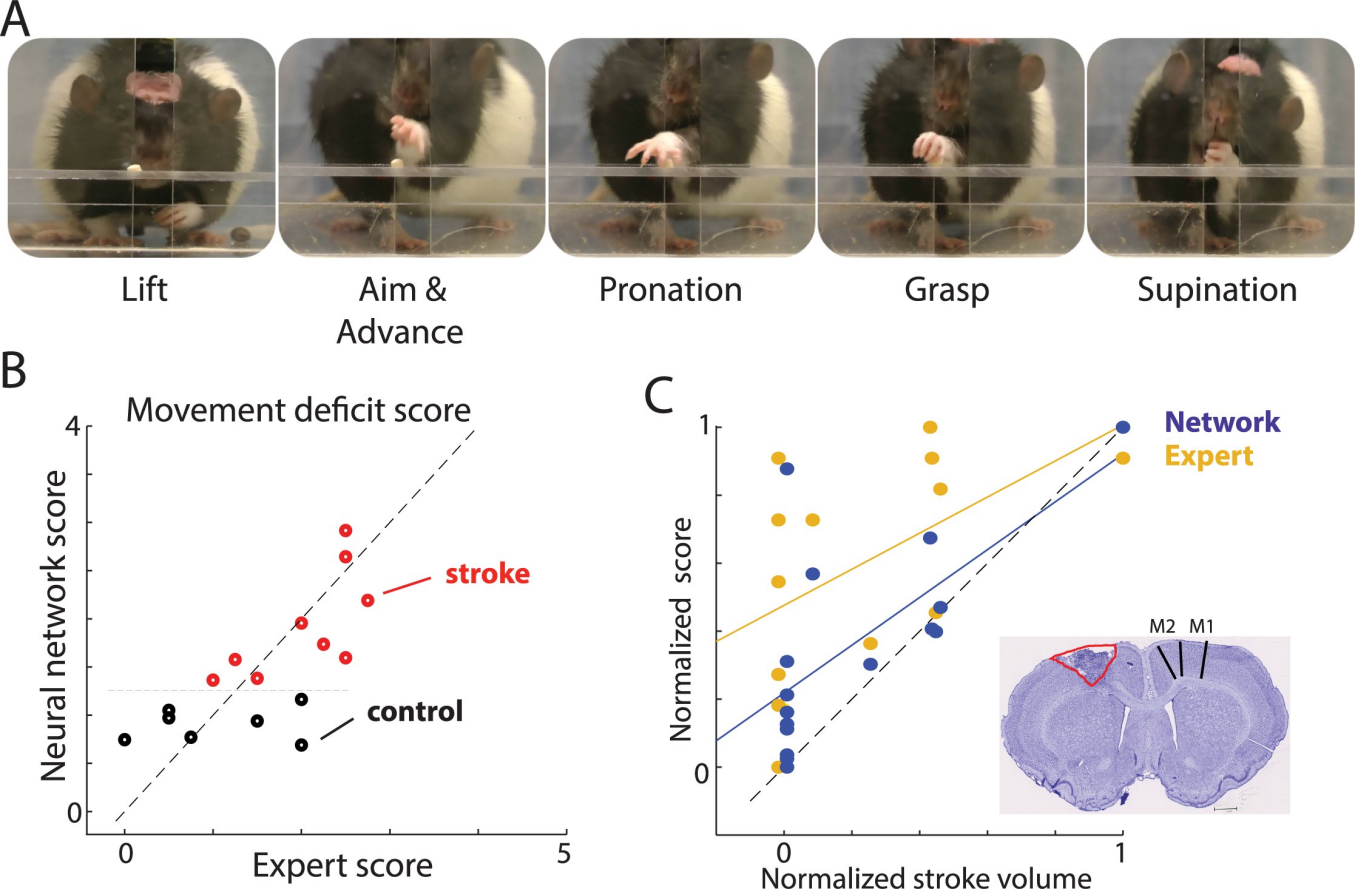
Automated scoring of movement deficits in the SPRT. (A) Video frames showing selected movement elements in the task. (B) Scatterplot of corresponding network and expert scores. Each circle denotes averaged score for a single rat. Note that stroke (red) versus control (black) could be separated along the network score (y-axis) but not along x-axis corresponding to the expert scores. (C) Scatterplot of stroke volume and corresponding scores by the network (blue) and human expert (yellow). The distribution of blue points closer to the identity line (dashed) indicates that network scores are more strongly correlated with stroke lesion volume than were the expert scores. Inset shows a representative histological image from a rat with a lesion (infarct area outlined; extent of M1 and M2 is denoted by lines in the intact hemisphere). Lesion volume and movement scores were normalized between 0 and 1 in order to directly compare both scores. Each dot represents the average score for one rat, and solid lines show linear regressions (blue: network score; yellow: expert score). The distribution of blue dots closer to the identity line (dashed) shows that the network scores better predict lesion volume in this dataset. The sample network and data on which this figure is based are available at github.com/hardeepsryait/behaviour_net. M1, primary motor area; M2, secondary motor area; SPRT, single-pellet reaching task.

To study motor deficits in stroke rats, we used a single-pellet reaching task (SPRT). Rats were individually placed in a Plexiglas chamber as previously described [3,40] and were trained to reach through an opening to retrieve sucrose pellets (45 mg) located in an indentation on a shelf attached to the front of the chamber (Fig 2A). A rat uses a single limb to reach through the opening and grasp a food item for eating, and behavior is video recorded from a frontal view. For each video clip, an expert scored the reaching movements using a standard scoring procedure to assess seven separate forelimb movement elements that compose a reach (Fig 2A). Each movement element (e.g., hand lift, aim, grasp) was scored using a scale: abnormal (1 point), partially abnormal (0.5 point), or within normal range (0 points) (Methods). Each movement element was scored independently, and the sum of those scores provides the behavioral measure of stroke severity [3]. The network was trained to reproduce the cumulative expert score for each reaching trial. For all predictions, we used “leave-one-rat-out” cross-validation, in which the predicted animal was excluded from the training dataset (Methods). The correlation between the average network score for each rat and the expert score was r = 0.71 (*p* = 0.002; Fig 2B), showing that the network can reproduce the expert score.

To determine whether the network scoring was within the variability range of human scorers, three other researchers (trained by the expert) independently rescored all the videos. The expert was IQW, with decades of expertise in behavior analyses, who developed this scoring system. His scoring was compared to scoring of three researchers: #1 (JF), a researcher with over 10 years of experience with behavioral scoring; #2 (HR), a researcher with 1 year of behavioral scoring experience; and #3 (SL), an undergraduate student with two semesters of scoring experience. For each rat, we measured the absolute value of the difference between the average scores of the expert and one of each researcher. The average difference across rats between the expert and other researcher scores was as follows: researcher #1 = 0.63 ± 0.09 SEM; researcher #2 = 0.77 ± 0.17 SEM; researcher #3 = 0.51 ± 0.1 SEM (S1 Fig). For comparison, the difference between the expert and network scores was 0.49 ± 0.08 SEM. Using the paired *t* test, we found that the discrepancy between expert and network scores was not statistically different from the discrepancy between expert and other researcher scores (S1 Fig). This shows that our network scores were within the variability range of trained humans.

Interestingly, our network scores were more correlated with the experimental group category (control versus stroke) than were the expert scores, although group information was not given to the network (r _Network-Group_ = 0.78, *p* = 0.0003; r _Expert-Group_ = 0.6, *p* = 0.015; see separation of red and black circles only along the y-axis in Fig 2B). Moreover, the network scores were better correlated with lesion volume than were the expert scores (r _Network-Lesion vol_ = 0.67, *p* = 0.004; r _Expert-Lesion vol_ = 0.5; *p =* 0.05; Fig 2C). To examine whether the network scores were significantly better than the expert scores in estimating lesion volume, we normalized the network and expert scores between 0 and 1 and compared them to lesion volumes, which were also normalized between 0 and 1. The network scores were significantly closer to the normalized lesion volume than were the expert scores (Wilcoxon signed rank test *p =* 0.0013, S2 Fig). The use of z-score normalization instead of 0–1 normalization resulted in the same conclusion. These results suggest that although the network was trained only to reproduce the expert scores, it did so by finding additional movement features that provided information about the stroke impairment (see following sections for further evidence).

The network was also able to accurately reproduce changes in movement deficit scores across days. For each rat, we calculated the average expert score on each recording day, and we correlated that score with the network score (the average correlation between the network and expert scores across days was r = 0.67). S3 Fig shows how the movement deficit score changed across days for each individual rat. The distribution of correlation coefficients (insert in S3 Fig) shows that for the majority of rats, the network tracked individual changes across days accurately (i.e., correlation coefficients approaching 1).

To test how the network’s performance depended on particular model parameters, we modified the network by changing the number of neurons and layers in the RNN, and we repeated the training and testing on the same data (S1 Table). The modified networks produced results consistent with those of the original network (average correlation coefficient between scores of original and modified networks: r = 0.93; every *p* < 0.0001; S4 Fig). The network also showed robustness to experimental variability of the video recording. Although on each video recording day, the camera, cage, and lighting were manually set in a predefined configuration, there were still noticeable variations in recording conditions across days (e.g., subtle differences in recording angle, distance, lighting, etc.). Training the network on videos only from 4 experimental days and predicting the rats’ scores on the remaining day confirmed that the network was generating reliable scores (average correlation coefficient between the expert score and the network score: r = 0.68, *p* < 0.01). Altogether, these results show that the network generalizes well to new rats and the variation in experimental conditions.

### Single-movement-element analyses

**Fig 3:**
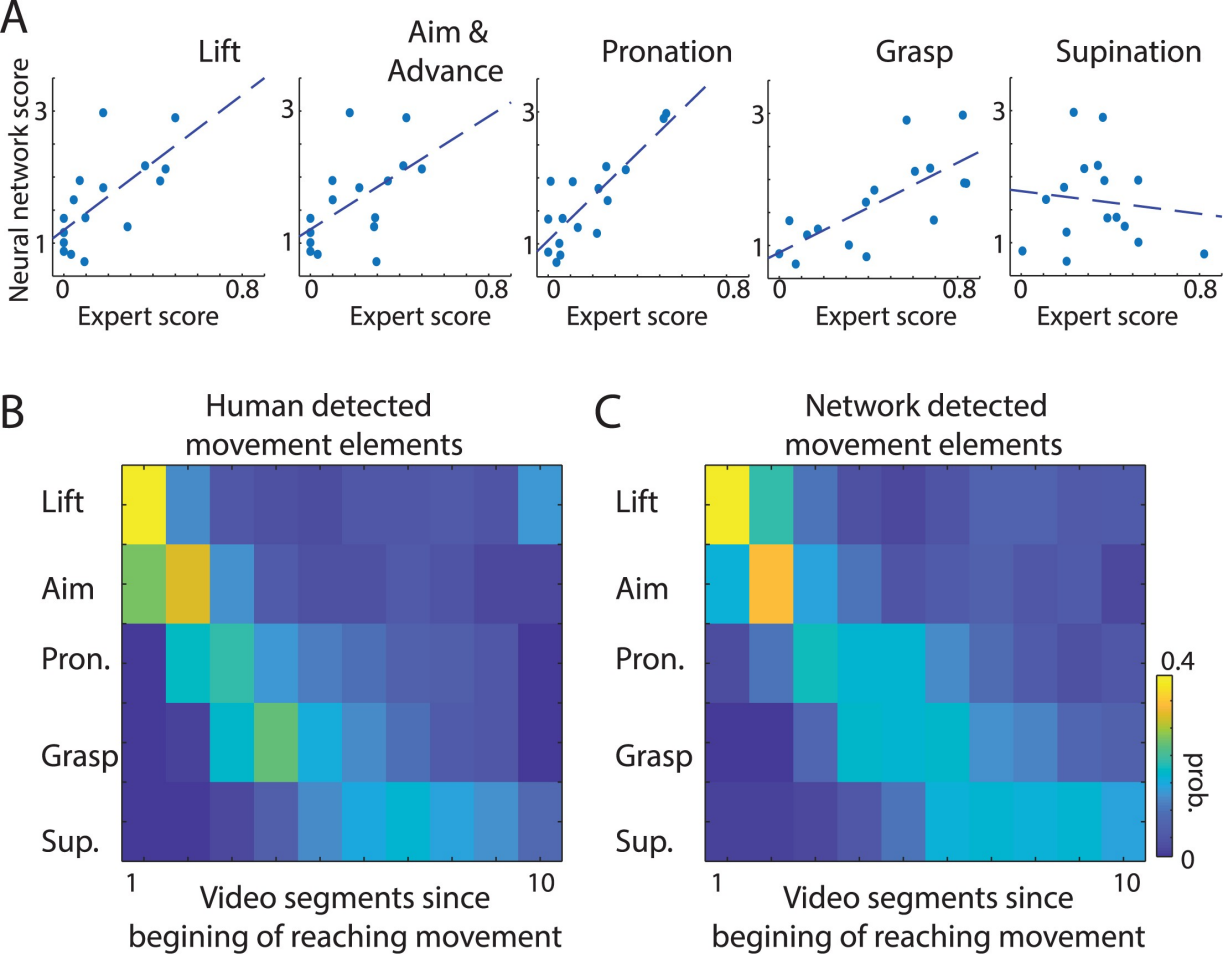
The network can learn to detect individual movement components with human-level accuracy. (A) The relation between network scores and expert scores for individual movement components. Each dot represents the average score for one rat, and dashed lines show linear regression. Network scores were significantly correlated with expert scores for almost all movement elements. (B, C) To directly test whether the network could learn to discriminate movement components in action clips, we retrained the network on video segments with labeled movement elements. Panels B and C show the probability (“prob.”) of detecting a particular movement element in a video clip. For visualization, video segments are aligned with respect to the beginning of a reaching movement. The high similarity between timing of movements defined by the expert (B) and the network (C) shows that the network can be used for automated segmentation of behavioral videos to identify specific movements. The sample network and data on which this figure is based are available at github.com/hardeepsryait/behaviour_net. Pron., pronation; Sup., supination.

Next, we investigated which movement elements were most informative for constructing the network’s movement deficit score. To estimate this, we correlated the network score with the expert score for each individual movement component (Fig 3A). Network scores were significantly correlated with all analyzed movement elements except for supination (r _Lift_ = 0.67, *p* = 0.005; r _Aim_ = 0.53, *p* = 0.03; r _Pron_ = 0.83, *p* = 0.0001; r _Grasp_ = 0.73, *p* = 0.001; r _Sup_ = −0.12, *p* = 0.66). To understand why supination did not correlate with network scores, we more closely examined our dataset, which revealed that two control rats had poor supination scores. Thus, the network correctly learned to “ignore” supination movement to derive the stroke disability score, because supination was not a consistent predictor for control rats (in behavioral analysis, experts often designate such scores as outliers). Therefore, our results should be taken as indication not that supination is not important for stroke evaluation but rather that it reflects the particular properties of the training dataset. Altogether, this suggests that the network, similarly to the expert, combined information from multiple movement elements to derive its scoring system.

To explicitly test whether the network could properly score individual movement elements, we trained the network to predict the expert scores of each movement element. For this, we added output neurons to the RNN that represented each individual movement element. The correlation between the expert and network scores for individual movement elements was r = 0.77, *p* < 0.001 (S5A Fig). This shows that the network is able to detect deficits in individual movement elements. Moreover, we tested how well the stroke volume could be predicted from the weighted combination of individual movement scores, rather than from the simple sum of individual movements’ scores. Multivariate linear regression showed that stroke volume was again better predicted from network scores than from expert scores (r _Network_ = 0.74, *p* < 0.01; r _Expert_ = 0.62, *p* < 0.01; (S5B Fig).

Automatically detecting instances of specific movement elements or postures can be highly useful for detailed behavioral analyses. Therefore, we asked whether the network could be trained to correctly classify different movement components in continuous videos. For this, we retrained the last layers of the network (RNN part in Fig 1) on video clips corresponding to separated movement components (each clip consisted of seven consecutive frames; Methods). Next, we tested the network on videos that were divided into video segments of seven frames. The correlation between the probability distribution of human and network labeling of movement classes was r = 0.89, *p* < 0.001 (i.e., correlation between Fig 3B and 3C). The average accuracy when comparing human and network classification in each individual video segment was 80.2% (S5 Fig). This demonstrates that the same network architecture can be used for automated segmentation of behavioral videos and for detecting specific movement components with human-level accuracy.

### Extracting information from the network

**Fig 4:**
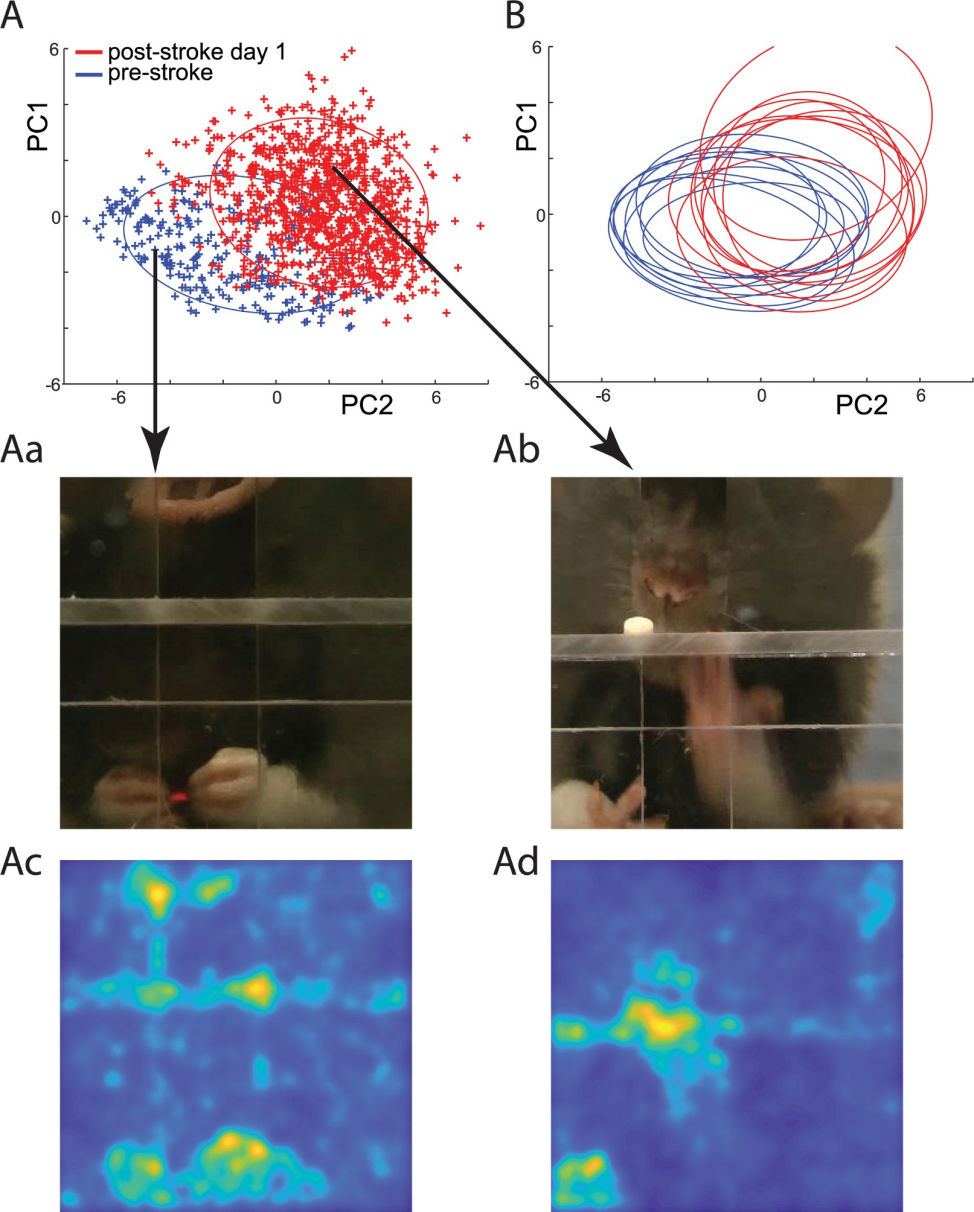
Extracting knowledge from the network to identify the movement elements most predictive of stroke severity. (A) Representation of video frames transformed into the internal feature space of the network (see Methods). Each point represents a single video fame. Blue points represent video frames from a single rat during trials obtained on the day before stroke. Red points represent video frames from trials obtained after stroke for the same rat. Blue and red ellipses outline distributions of points before and after the stroke, respectively. Note the disparity between distributions. For example, eating with both hands (Aa) was only observed before the stroke, whereas reaching for the food pellet with the mouth (Ab) was only observed after the stroke. Panels Ac and Ad illustrate the parts of frames in Aa and Ab that the network evaluated as being most important for its scoring decision. (B) Ellipses outline the distribution of points before the stroke (blue) and on day after the stroke (red) for each rat. Close overlap of the red ellipses indicates that features predictive of stroke found by the network were consistent across rats. The sample network and data on which this figure is based are available at github.com/hardeepsryait/behaviour_net. PC, principal component.

Considering that the network scores produced a higher correlation with stroke lesion volume than did expert scores (Fig 2B and 2C), we investigated which movement features were the most informative for the network scoring. For this, we applied recently developed tools for knowledge extraction from deep neural networks [38,39]. First, we identified which features extracted from video frames were contributing most to the score by the RNN (features marked in red in the middle part of Fig 1; Methods). Out of the 2,048 features, we selected about 200 with the highest contribution and then performed principal component analysis (PCA) on those selected features. Thus, each original video frame was transformed to a low-dimensional PCA space of the most-informative features. For example, Fig 4A shows points in PCA space corresponding to video frames recorded before and after the stroke for a single rat. The disparity between clusters corresponding to different days shows that there are a large number of frames with features specific only to the normal or to the stroke condition. For instance, frames showing rats eating with both hands were only present before stroke (Fig 4Aa), and frames showing rats trying to reach for food with the mouth instead of the hand were only present after the stroke (Fig 4Ab). We further asked the network to identify the parts of each frame that were used for the network decision (Methods). For example, for the video frames shown in Fig 4Aa and 4Ab, this confirmed that the network was mainly using hand and mouth features in those frames to calculate the motor-disability score (Fig 4Ac and 4Ad). The differences found by the network in reaching behavior pre- versus poststroke were consistent across rats. This is illustrated in Fig 4B, in which each ellipse outlines the distribution for pre- and poststroke day for each rat. Thus, by using the network representation, we could identify which features of the behavior that were the most indicative of cortical stroke.

### Discovering movement elements based on the internal network representation

**Fig 5:**
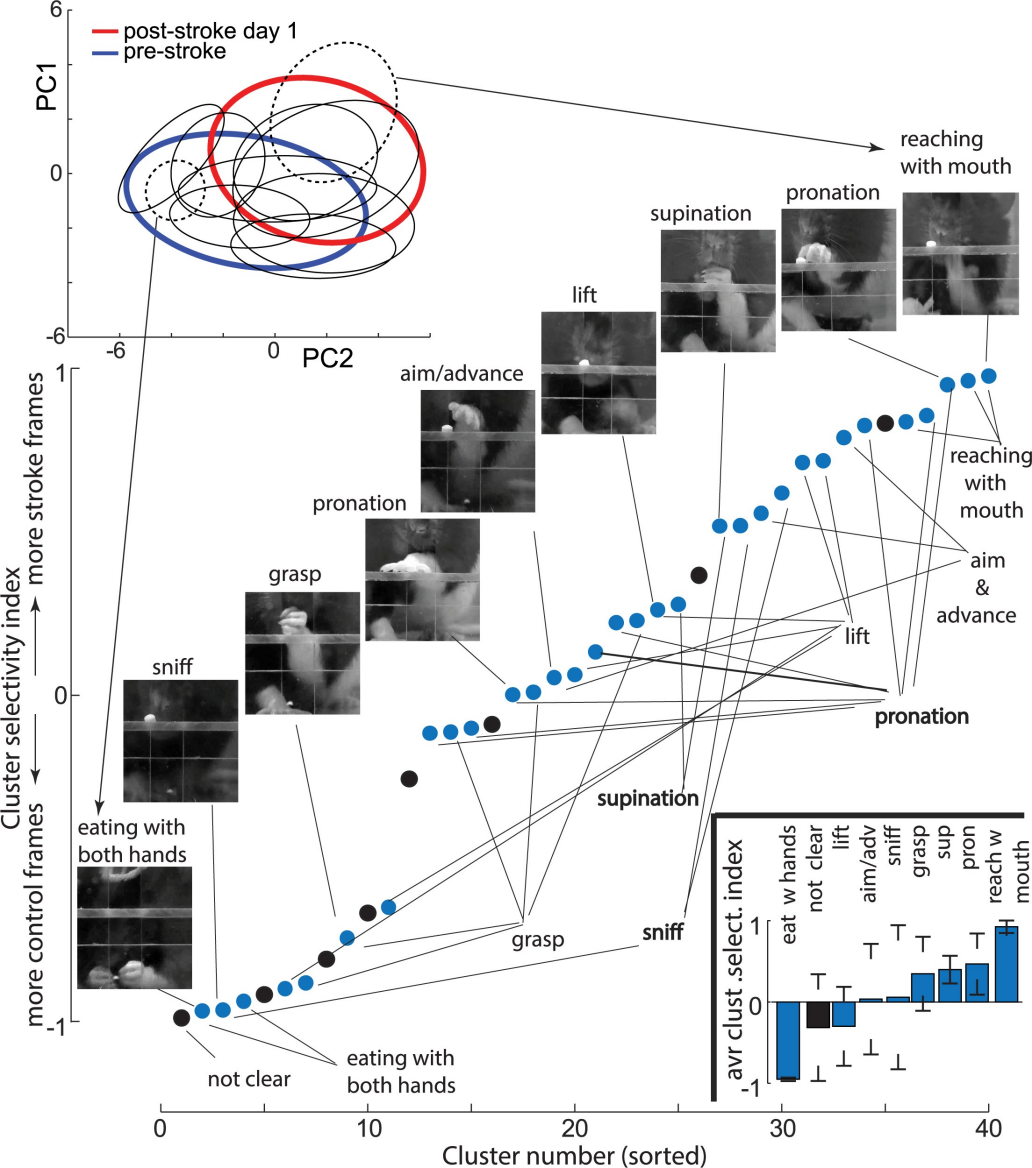
The clustering of the network feature space revealed movement elements specific only to the stroke or the control condition. (Top insert) Blue and red ellipses outline the distribution of points in feature space of the network before and after the stroke, respectively (the same as in Fig 4A). Black ellipses outline subclusters corresponding to individual movement subcomponents. For visualization clarity, only 10 subclusters out of 40 are shown. Dashed ellipses indicate clusters most selective for the stroke and the control categories and arrows point to sample frames from those clusters. Note that clustering was done using the first seven PCs of the network features; thus, subclusters appear to overlap in this 2D projection. (Main panel) Each point represents cluster selectivity by expressing the fraction of frames from stroke versus control rats in each subcluster (see Results). Labels below denote the movement category assigned to subclusters, and images above show representative frames from corresponding subclusters. Points in black denote a “not clear” clusters category. The bottom insert shows the average cluster selectivity index (“avr clust select. index”) for each movement category. Error bars denote standard deviation. The sample network and data on which this figure is based are available at github.com/hardeepsryait/behaviour_net. adv, advance; PC, principal component; pron, pronation; sup, supination.

To better understand the relationship between the internal network representation and the movement components, we divided points in the network feature space into disjoined clusters (Fig 5 top insert). We used data from a day before and a day after the stroke and applied an unsupervised k-means clustering to divide it into 40 subclusters (changing the number of subclusters between 20 and 60 did not affect the presented conclusions; S6 Fig). After closer examination of the resulting subclusters, we found that most subclusters could be clearly assigned to one of the movement categories: lift, aim and advance, pronation, grasp, supination, sniffing, reaching for food pellet with a mouth, and eating with both hands. Thus, for each subcluster, we assigned one of the above categories based on the examination of eight frames closest to the subcluster center, which was evaluated by two researchers. If four or more frames were judged to be in the same category, then that category was assigned to the subcluster. Otherwise, we assigned a “not clear” category, meaning that this subcluster contained frames from a variety of movement elements. There were also off-task frames (e.g., rearing or a rat walking away), but these types of frames did not form consistent subclusters and were thus assigned to the “not clear” category. For example, the dashed ellipses in Fig 5 outline subclusters corresponding to movement components described in Fig 4Aa and 4Ab, which were characteristic for control and stroke conditions, respectively.

To quantify the selectivity of the clusters for the stroke versus control group, we counted in each subcluster the number of frames from each treatment group. Specifically, we devised a cluster selectivity index as (# of stroke frames − # of control frames)/(# of stroke frames + # of control frames), which has values bound between −1 and 1. For example, a cluster selectivity index = 1 means that this subcluster contains only frames from videos of stroke rats. The cluster selectivity index = 0 means that a subcluster has equal number of frames form videos of stroke rats and control rats. The category assignments for all subclusters, sorted by the cluster selectivity index, is shown in Fig 5. Most movement elements—for example, “lift”—had multiple subclusters, with some subclusters containing mostly control frames and other subclusters containing mostly frames from the stroke group. This could be interpreted as a difference in how that movement element is executed in controls versus stroke rats, which is consistent with the main premise behind an expert scoring system [3]. However, consistent with observations shown in Fig 4, we also found two distinct movement elements: eating with both hands and reaching for a food pellet with the mouth, which almost exclusively had frames only from control or stroke rats, respectively. To quantify these observations, we calculated the average cluster selectivity index for each movement category: lift = 0.03 ± 0.67 SD, aim and advance = 0.46 ± 0.37 SD, pronation = 0.34 ± 0.45, grasp = −0.29 ± 0.48, supination = 0.39 ± 0.17 SD, sniff = 0.06 ± 0.88, not clear = −0.31 ± 0.65, reaching with mouth = 0.92 ± 0.07 SD, eating with hands = −0.95 ± 0.02 SD (see bottom insert in Fig 5). This shows how our data-driven approach can help to discover the most-informative movement elements. Those movement elements then can be used as the basis for designing improved behavioral scoring systems for neurological disorders.

### Changes in individual movement elements during stroke rehabilitation

**Fig 6:**
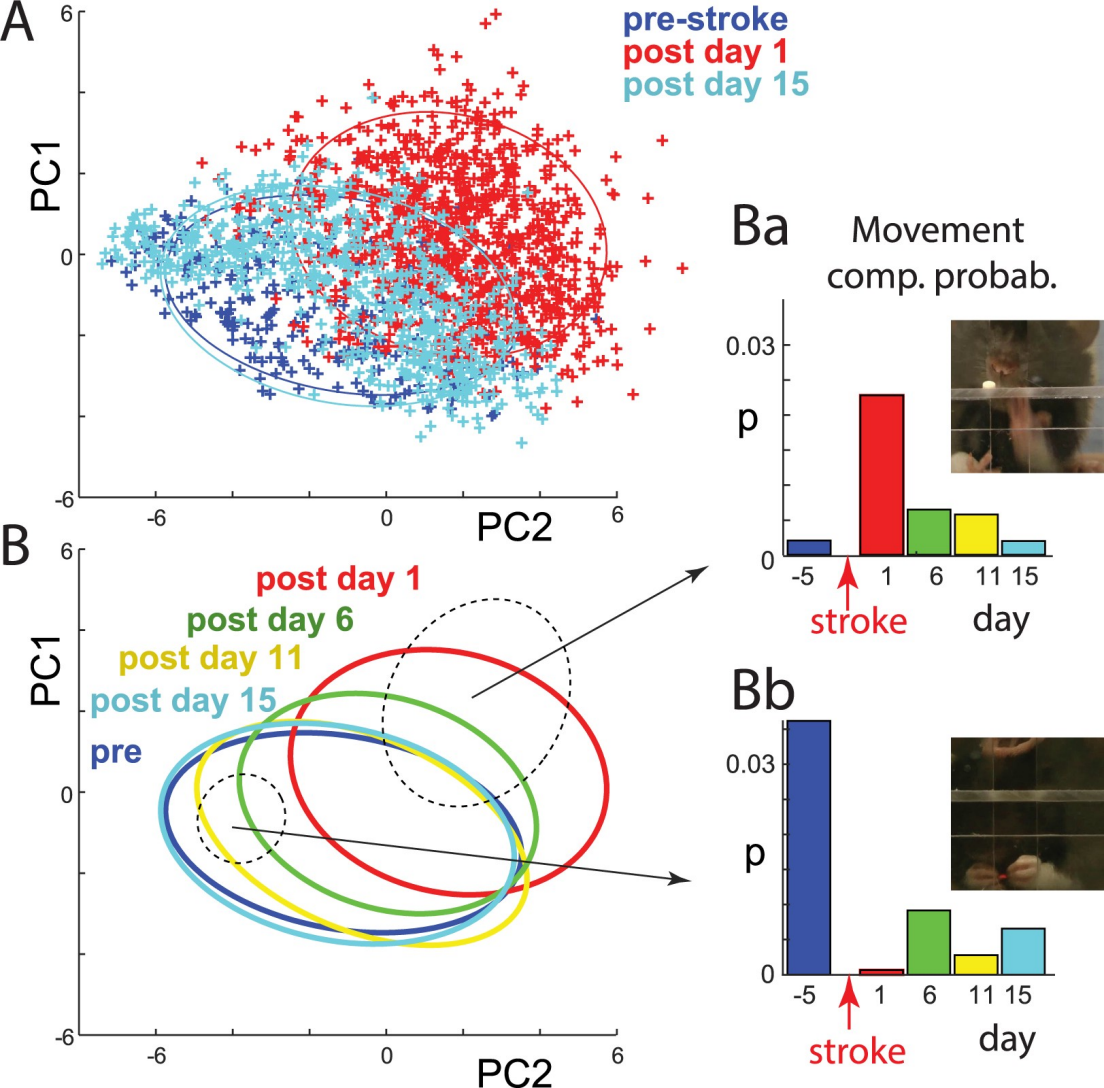
Quantifying changes in individual movement components during stroke recovery using the internal network representation. (A) Representation of video frames in the internal feature space of the network, as in Fig 4A, but with added points from day 15 after stroke (light blue). Note that points on day 15 shift toward prestroke (dark blue) values, indicating movement recovery. (B) Ellipses outlining the distribution of points before stroke and for all filming days after stroke. Note the gradual shift of the poststroke distributions toward prestroke space. Dashed ellipses illustrate sample subclusters representing single movement components. (Ba and Bb) Probability of points falling within a given subcluster across days. For example, the high red bar in Ba shows that this movement component was mostly present on day 1 poststroke. The sample network and data on which this figure is based are available at github.com/hardeepsryait/behaviour_net. Movement comp. prob., movement component probability; PC, principal component.

Plotting video frames in principal component (PC) space of the network representation revealed that in the days following stroke, movement components started returning to prestroke values (Fig 6A and 6B). Interestingly, data clustering, described above (Fig 5), allowed us to analyze poststroke changes for each separate movement element. For this, we calculated the number of video frames within each cluster separately for each experimental day (the number of frames in each cluster was normalized by the number of frames recorded that day; thus, it is expressed as a probability: *p*). For instance, Fig 6Ba shows that before stroke, it was unlikely that a rat would try to reach for a food pellet with its mouth. After stroke, the probability of that movement increased and then reverted toward the control level as rehabilitation progressed. For the subcluster corresponding to a rat eating with both hands, this movement almost completely disappeared immediately following stroke, and it shows very little recovery in the following days (Fig 6Bb). Changes across days during stroke recovery for all subclusters are summarized in S7 Fig. Importantly, these analyses allowed us to quantify stroke recovery (the return of normal or movement elements, e.g., Fig 6Bb) versus compensation (the appearance of new movements, e.g., Fig 6Ba), which can be important for improving monitoring the effects of rehabilitation.

### Illustrating complex movement trajectories using the internal network representation

**Fig 7:**
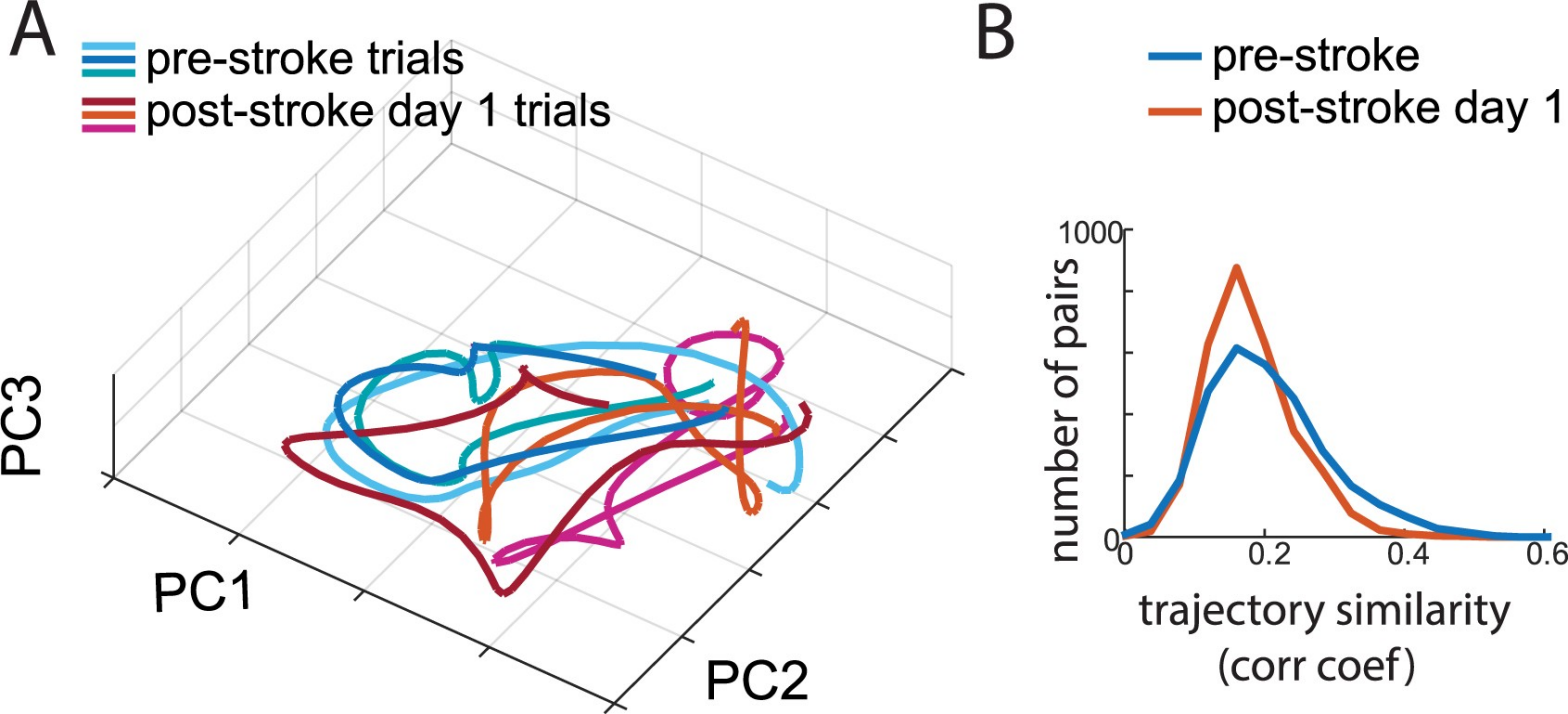
Movement trajectories encoded by the internal network representation are more variable after stroke. (A) Movement trajectories for the three most similar trials before stroke (blue shades) and the three most similar trials after stroke (red shades) for the same rat. Coordinates correspond to the first three PCs of the internal network representation. (B) Distribution of correlation coefficients (“corr coef”) between pairs of trajectories for the day before stroke (blue) and the day after stroke (red). The sample network and data on which this figure is based are available at github.com/hardeepsryait/behaviour_net. PC, principal component.

Typically, movement trajectories represent the sequential positions of a single body part in three spatial dimensions as a function of time. In contrast, the trajectory in the PCA space of internal network representation (Fig 7A) represents combinations of multiple body features that were the most informative in indicating stroke-related abnormalities of movement. This representation shows that after stroke, the behavioral trajectory becomes more variable. For quantification, we calculated cross-correlograms between pairs of trajectories (S8 Fig). We found that before stroke, there were significantly more highly reproducible trajectories (*p* < 0.001, *t* test; Fig 7B). Moreover, the variability of the trajectories within a single session (consisting of 20 reaching trials) was significantly correlated with the overall movement deficit score (r = −0.41, *p* < 0.001).

### The network can derive expert-like scores from only categorical data (stroke = 1, control = 0)

**Fig 8:**
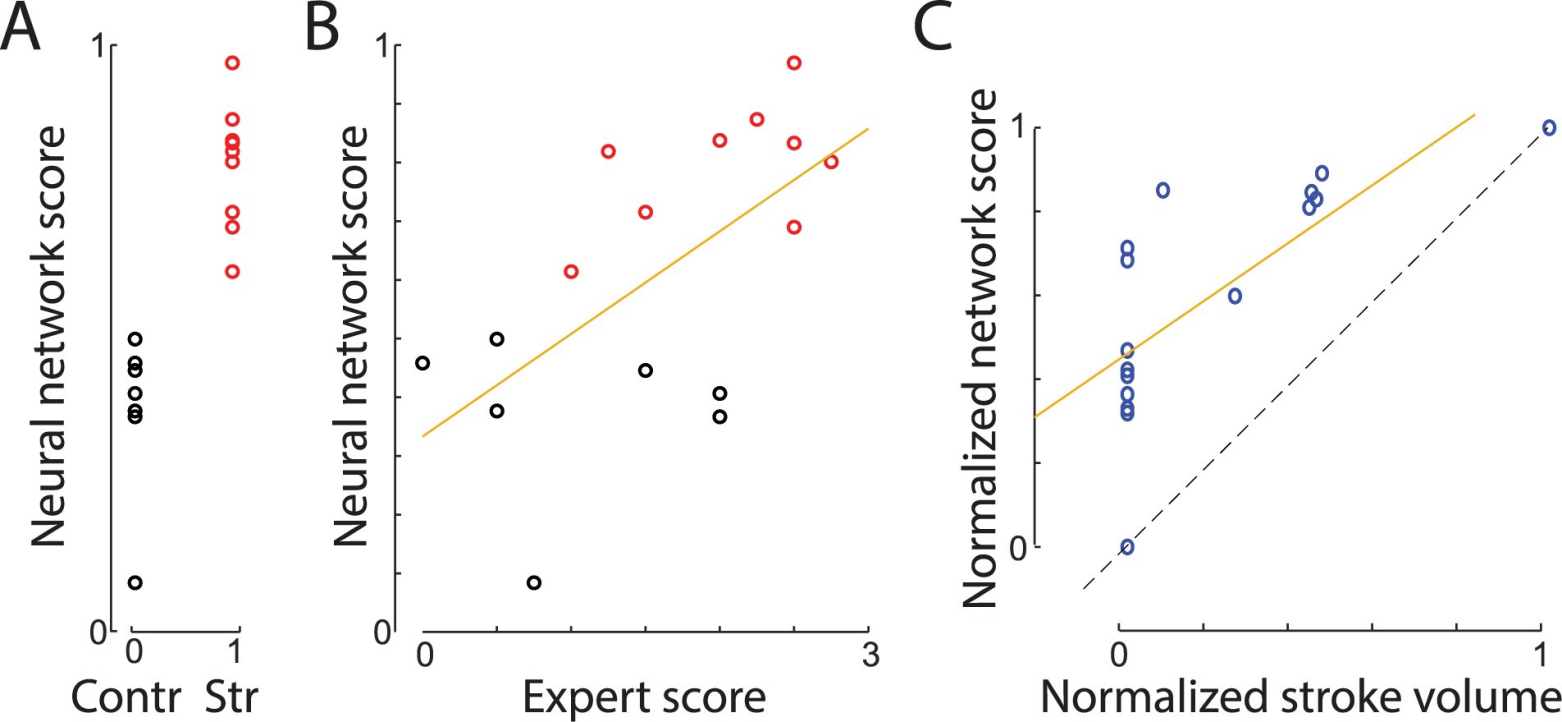
A network trained only to classify videos as stroke versus control derived a continuous expert-like score. (A) Neural network scores versus group category used for the training. Each circle denotes averaged score for a single rat (stroke [“Str”] = red, control [“Contr”] = black). (B) Relation between that network scores and the expert scores. The regression line is shown in yellow. (C) Network scores were also predictive of stroke volume, even though this information and human-based scores were made available to the network. Stroke volume and movement scores were normalized between 0 and 1 in order to directly compare both scores. The sample network and data on which this figure is based are available at github.com/hardeepsryait/behaviour_net.

Creating a dataset with expert scores to train the network can be time consuming. For example, to score 692 reaching trials used here, it took about 60 hours for one trained person (approximately 5 minutes per trial). To eliminate such a laborious human scoring requirement, we provided the network only with the class information (stroke versus control) for each trial. The aim was to determine whether our regression network, if trained only on categorical labels (stroke = 1; control = 0), could then estimate the level of stroke impairment. The network had two output neurons (n0 and n1) corresponding to stroke and control class. However, when presented with the test example, neurons n0 and n1 usually had values between 0 and 1, reflecting how “certain” the network was that a presented trial belonged to stroke or control category respectively. Therefore, we defined the network score to be the average vote of both neurons: Nsc = [n0 + (1 − n1)]/2. The network learned to discriminate the stroke versus control groups with 100% accuracy (Fig 8A). Network scores were also well correlated with the expert scores (r = 0.61; *p* = 0.012). The discrepancy between the network and the expert scores (1.04 ± 0.16 SEM) was not statistically distinguishable from the discrepancy between the expert and other trained researchers (*p*_researcher#1_ = 0.03, *p*_researcher#2_ = 0.15, *p*_researcher#3_ = 0.04), showing that training with only categorical information can produce movement scoring at or close to human accuracy. The network scores were also highly correlated with stroke size (r = 0.73, *p* = 0.0014; Fig 8C), which provides additional support for the effectiveness of this approach. Altogether, these results show that training the network only on a stroke versus control category can produce movement scoring similar to the scoring developed by human experts. This provides proof of concept that the presented approach can provide easy-to-implement, data-driven behavioral scoring when expert scoring is unavailable or impractical.

### Training the network on stroke size data converges to a similar solution as training on expert scores

**Fig 9:**
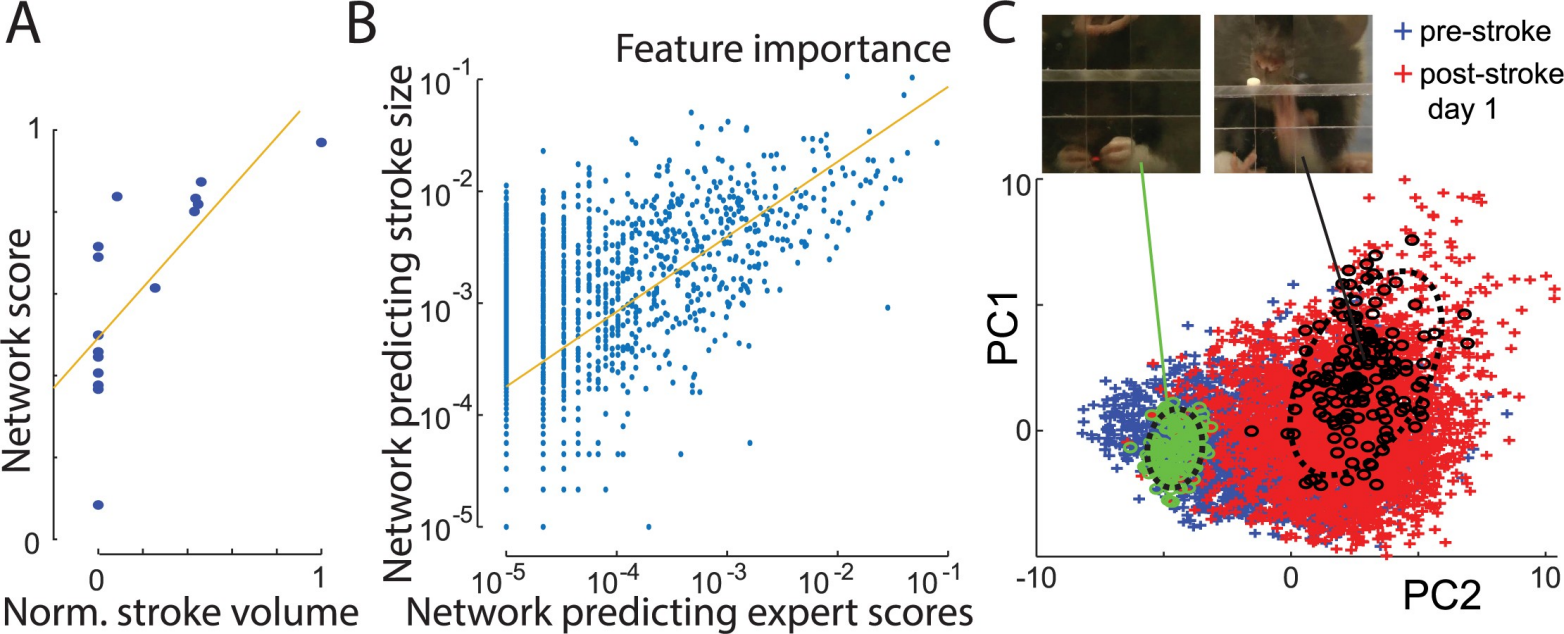
A network trained to predict stroke size discovered the same most informative movement features as the network trained to predict expert scores. (A) Network predictions of stroke lesion volume (normalized [“Norm.”] between 0 and 1). The line shows linear regression. (B) Importance of movement features as determined by the network trained on stroke size (y-axis) and the network trained on expert scores (x-axis). Each point represents one of 2,048 features from the output of the ConvNet (Fig 1). (C) Representation of video frames in internal feature space of the network trained to predict stroke volume (see Fig 4A for description). Green and black points correspond to frames identified in previous analyses (see Fig 5) as belonging to reaching with the mouth and eating with both hands (outlined with dashed ellipses). The similar location of those clusters to the corresponding ones in Fig 5 exemplifies the discovery of similar feature importance by both networks. The sample network and data on which this figure is based are available at github.com/hardeepsryait/behaviour_net. ConvNet, convolutional network; PC, principal component.

Stroke size calculated from brain slices provides an anatomical measure of stroke severity. However, this measure may not perfectly correlate with behavioral deficits [41]. This is because strokes of similar size and location may result in a different degree of impairment among animals, because of variability between brains and its vasculature. Nevertheless, lesion size is a highly relevant measure of stroke severity. Accordingly, we trained our network to predict stroke size from the same videos of rats performing the reaching task. The correlation between stroke size and network predictions was r = 0.86, *p* < 0.001 (Fig 9A). Scores generated by this network were also highly correlated with scores of the first network trained to reproduce expert scores (r = 0.73, *p* = 0.0013). This suggests that networks trained to predict stroke size and those trained to predict expert scoring converged to similar solutions.

To investigate which features were the most important for network predictions, we used the analyses described previously (Fig 1). For all our networks, we used exactly the same ConvNet part, and only the RNN part was modified. Therefore, we analyzed which output features of ConvNet were the most important for each RNN network. Using ϵ-layer-wise relevance propagation (eLRP) algorithm (Methods), we calculated the importance of each of the 2,048 features and averaged them across all videos. We found that the most informative features for the network’s prediction of stroke size were also the most important features for the network trained to reproduce expert scores (Fig 9B). The correlation coefficient between feature importance for two networks was r_NetStrokeSize-NetExpertScores_ = 0.62, *p* < 0.0001 (values of feature importance varied over orders of magnitude; thus, all values were log transformed before calculating a correlation coefficient). Similarly, comparing feature importance between all pairs of the networks presented here (trained to predict expert scores, stroke versus control, stroke size, movement element impairments) also resulted in highly significant correlations (all *p* < 0.0001; average r = 0.61 ± 0.05 SEM). This shows that regardless of exact task, all networks picked similar features for stroke-related predictions.

To further investigate similarities between movement features used by our networks, we again applied PCA analyses to features that were the most informative for network predictions of stroke size. Consistent with analyses in Fig 4A, we selected the 200 features that had the highest contribution to the network decision. Plotting the results in PCA space again revealed differences between the video frames from rats before and after stroke (Fig 9C). To check whether the positions of clusters corresponding to individual movement elements were also similar between networks, we marked (in green) points corresponding to frames classified before as belonging to the subcluster “eating with both hands.” Similarly, we marked in black points belonging to the “eating with mouth” subcluster, as defined by the k-means algorithm described in the previous section (compare Fig 9C with top insert in Fig 5). This shows that the subclusters most discriminative between stroke and control groups for the network trained to reproduce expert scores were in similar disjoined parts of the feature space for the network trained to predict stroke size. We suggest the following analogy: To predict the age of trees, a network may discover that width and height are the most predictive features. Similarly, if a network is trained on a classification problem to discriminate old versus young trees, it would discover that the same features are the most predictive (width and height), resulting in similar PCA projections. Moreover, scores generated by the network trained on stroke size also significantly correlated with expert scoring (r = 0.51, *p* = 0.043). Altogether, these results demonstrate that networks trained on different tasks related to stroke scoring find consistent movement features predictive of stroke severity. This is important because it shows that the network does not need to be trained with expert scores to discover movement features that are the most predictive of stroke impairments.

### Comparisons of our approach to other methods used for behavioral analyses

Considering that some movement elements can significantly differ between stroke and control conditions, it may be expected that simpler methods than deep neural networks could also predict expert scores and stroke severity. To test this, we applied PCA to all combined video frames. We took the first 20 PCs to represent each frame (explaining 71% of variance; S9A Fig), and we applied least-squares regression to predict expert scores (all frames in the same video clip corresponding to a single trial were assigned the same score to predict). Using this simple linear approach, the correlation between expert scores and predicted scores was not significant (r = −0.1 *p* = 0.69). To investigate this further, we used t-distributed stochastic neighbor embedding (t-SNE) [42] to visualize all 20 PCA components in 2D space (S9B Fig). We found that small changes in video procedures—e.g., camera angle—caused a large change in PCA scores. For example, subtle shifts of the camera during a filming day caused large variability in the PCA space (S9B Fig). In contrast to the ConvNet, which can extract features invariant to spatial shifts, PCA features cannot be used to easily examine differences between stroke and control rats without careful realignment and rescaling of all frames.

To test more directly how informative PCA features are as compared to ConvNet features, we took 2,048 PCs as a description of each frame (99.3% explained variance). Next, we combined all frames from a single trial in an array, and we used the RNN network instead of the least squares for predicting expert scores. Thus, we replaced ConvNet features in our network (Fig 1) with PCA features. This resulted in improved predictions of expert scores over the least-squares method (r = 0.48, *p* = 0.06); however, using PCA was still markedly worse than using ConvNet (compare S9C Fig to Fig 2B).

Recently, other methods based on deep neural networks have been developed for automated analyses of animal behavior [43,44]. However, those methods are designed to track body parts rather than directly predict movement deficits. Specifically, these methods provide x- and y-coordinates of selected body parts, which then need to be interpreted; i.e., to predict motor deficits, additional analyses are required. Thus, our method offers an alternative to those approaches, as our network can directly extract disease-related movement features.

To test whether x- and y-coordinates could provide better features than the ConvNet for predicting expert scores, we used DeepLabCut [44] to track the position of the nose and of two fingers and the wrist on each forepaw (S10A Fig). As a result, each video frame was represented by x- and y-position values of seven marked body parts and by seven additional values representing the DeepLabCut confidence of estimates of each point. All points corresponding to frames from one trial were combined as one input to the RNN (similarly as ConvNet feature in Fig 1). The correlation between predicted and actual expert scores was r = 0.53, *p* = 0.036 (S10B Fig). This suggests that ConvNet features, selected in a data-driven way, can outperform human-selected features (marks on body parts) to predict motor deficits. Different selection of body parts may result in improved performance; however, note that reliably identifying joints on a furry animal with pliable skin is sometimes difficult. Therefore, the advantage of our network is that it can directly predict movement deficits from raw videos and does not require human selection of body parts to predict movement scores.

### Network performance on other behavioral tasks

**Fig 10:**
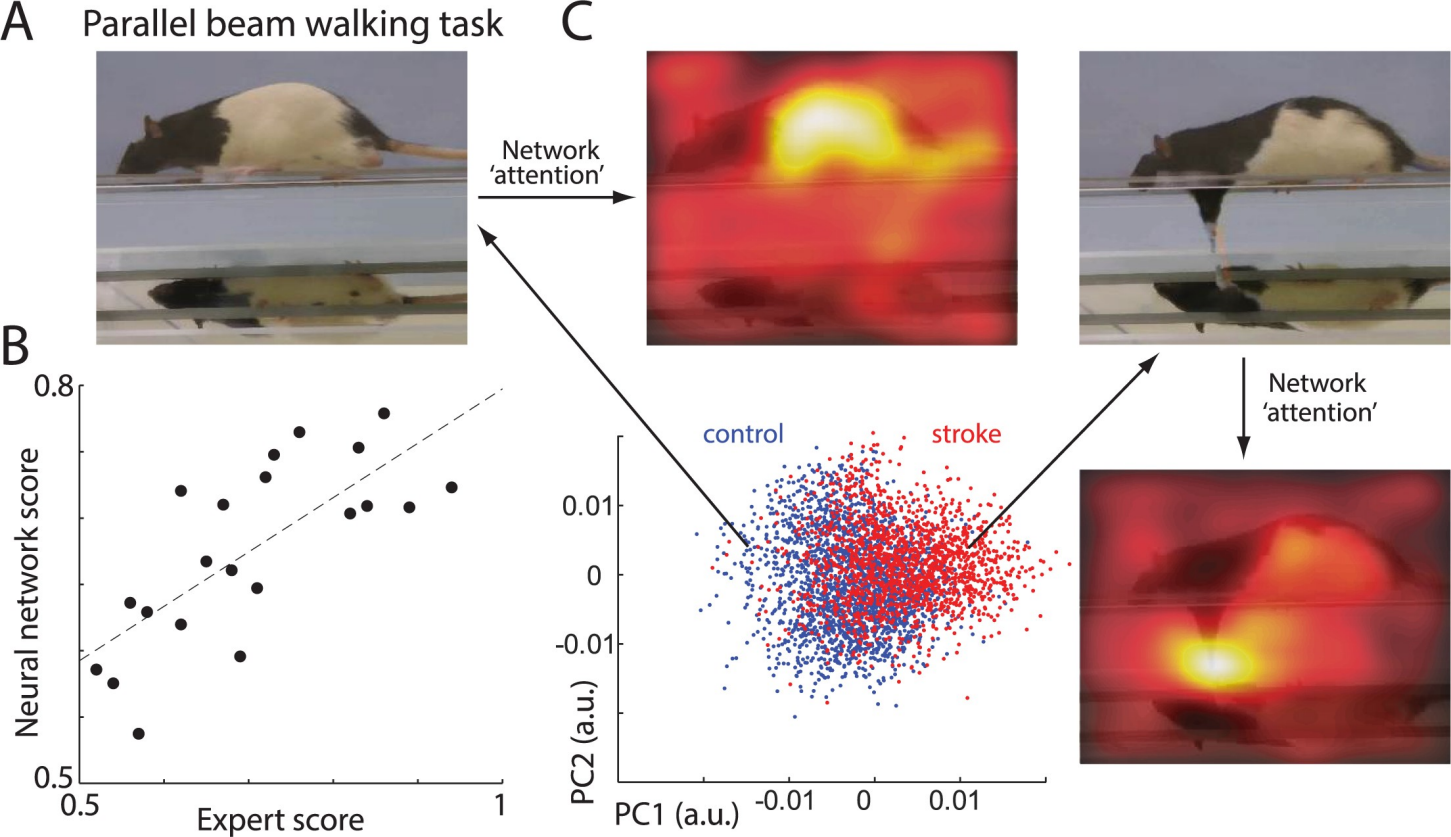
Network validation on different behavioral tasks. (A) Sample frame showing a rat on the parallel-beam-walking task. Note the mirror below the rat is showing an additional view of paw placement. (B) Relationship between expert and network scoring. Each dot represents the average score for a single rat. (C) Representation of video frames transformed into the internal feature space of the network. Each point represents a single video fame. Blue points represent video frames from control rats and red points from stroke rats. For picture clarity, only 20% of randomly selected points are shown. Long arrows point to sample video frames from control and stroke groups. Short arrows point to “attention” maps superimposed on frames: parts of frames most informative for network decision (marked in lighter colors). It indicates that similar to experts, the network uses foot slips to score stroke deficits (micro symptoms), but it also discovered that body posture and/or speed (macro symptoms) improves scoring. The sample network on which this figure is based is available at github.com/hardeepsryait/behaviour_net. a.u., arbitrary units; PC, principal component.

The same network can also be trained to score and analyze a whole spectrum of different behavioral tasks (S11 Fig). As an example, we trained our network to reproduce expert scoring for rats performing the parallel-beam-walking task (Fig 10A). Here, fine inaccuracies in paw placement and paw slips were counted to provide a measure of movement impairment after stroke [13–17] (Methods). The network scoring significantly correlated with the expert score (r = 0.74, *p* = 0.0001; Fig 10B), showing that the network successfully learned to score this task. To identify which movement features the network was using to score deficits, we repeated the same analyses as presented in Fig 4. Briefly: first, we used the eLRP method [38] to identify which features extracted by ConvNet contributed the most to the RNN output score. Those features were than projected to 2D space (Fig 10C). Here, to find PCs, we used partial least squares [45]. This method is similar to PCA, but its components are chosen to explain most variance between stroke versus control groups, thus allowing us to more easily identify distinctive frames for each group. For example, in Fig 10C, most points on the left side correspond to the control animals, and most points on the right side represent frames from stroke animals. Representative frames for each group are marked with arrows. For each video frame, we also extracted information about which parts of a frame were used for the network decision (see Fig 4 and Methods for details). Loosely speaking, this illustrates where the network is paying most “attention” in solving the scoring task. For control animals, the network mostly focused on the center of rat. This suggests that the network may be using a simple speed or posture discrimination rather than details of foot placements, which are used by the human expert scoring system. For example, we noted that posture is different between stroke and control rats. Stroke animals tend to have a lower center of mass and hug the beam (similar to an elderly person who walks with a bent back and prefers to be close to the railing so as to better hold it). Consequently, the control rat had a more normal convex back, whereas the stroke rat often displayed a concave (lordosis) posture. Thus, the network appears to have discovered that posture can help to score stroke deficits. Nevertheless, when the rat’s foot slipped, the network was clearly detecting that by using the part of image with the forepaw for its decision (Fig 10C right side). Thus, similar to the SPRT, the network identified movement components (micro symptoms) used by experts to score behavioral deficits (food slips), but it also used indices of whole body action (mega symptoms) to make decisions.

## Discussion

Here, we describe a neural network that is trained to score the skilled reaching movements in control rats and in rats with a motor cortex stroke. The scores produced by the network and the scores produced by human evaluators were highly correlated. Both the network and the evaluators found that movement scores correlated with stroke size, but the network was more successful in predicting stroke size. Both the network and the experts found that performance was severely impaired on the first day after the lesion, and both found that scores improved but did not fully recover over 15 days of rehabilitation. The network also examined the spatial trajectories of the movements and found that the trajectories were more variable poststroke than prestroke. An analysis of how the network made decisions found that the network classified movements in a way that was similar to that of the evaluators and also used features of the movements that were not part of the expert scoring system.

We chose the SPRT to evaluate the network, because over the past 30 years, this task proved to be a sensitive behavioral test for identifying reaching impairments in animal models of stroke Huntington disease, Parkinson disease, and spinal injury and in patients with these conditions (for reviews, see [3,6,27,46–50]). Interestingly, the knowledge extraction from the network revealed that the standard movement components scored in this test (e.g., arm lift, pronation, grasp, etc.) were not the only ones informative of neurological deficits. The network additionally identified behaviors not included in this scale, including eating with both hands at the end of trial, reaching for pellet with the mouth, and postural differences that were different in control and stroke rats. This suggests that the network is able to use a wider range of information than that included by experts in a behavioral scoring system.

Similarly, differences in scoring strategy may be a reason for better correlation of network scores with stroke volume. Rating scales used to assess behavior are heuristic, designed to simplify diagnosis. The network, however, is not so constrained and thus may ignore single movement inaccuracies that are not predictive of stroke but attend to others that are. Moreover, human scores are designed to evaluate separate movement elements, whereas the network can additionally use temporal combinations of movement features (micro and macro symptom analyses), which may provide a better stroke predictor.

The neural network described here offers a state-of-the-art method for an automated scoring of complex behaviors. Such automated analyses provide multiple benefits: they can reduce time and personnel needed for scoring, which in turn can allow for more detailed and frequent behavioral testing. Moreover, they can improve scoring validity and reliability by eliminating interrater biases [51]. Usually, to automatically analyze behavior, tracking systems are used for which markers are attached to body parts (for review, see [52]). Interestingly, a deep neural network has recently been shown to successfully track “virtual” markers, wherein the virtual marker position is specified only on a subset of the video frames (training), and the network predicts the position of those markers in the rest of the video [44]. Moreover, computer science advancements allow for automated video analyses of variety of behaviors [53–64], which could be used to correlate changes in behavior with health deficits [63,65]. Nevertheless, an important point to note is that most of these methods rely on a human-defined selection of a subset of features (e.g., number and location of body markers, definition of behavioral states, etc.). Here, we demonstrate that a neural network can forego such assumptions and rather use a data-driven approach (e.g., training on categories or lesion volume). A similar data-driven approach is used by MotionMapper [62] and MoSeq [63] methods, which also do not require human-defined selection of features. However, those methods do require image preprocessing and proper image alignment. Moreover, after projecting videos to feature space using those methods, other methods must be applied to classify normal versus mutant animals. A key advantage of our methods is that by using ConvNet, it can work with raw images without need of preprocessing and without the sometimes difficult task of image alignment. This may prove useful for analysis of videos conducted outside of a laboratory and by nonexperts, such as home video recording of neurological symptoms. Furthermore, the RNN part of the network provides a powerful module for classifying spatiotemporal patterns to score behavior, thus offering a one-step solution for feature selection and predictions.

This method can have important implications for diagnostics. For example, clinicians presently score behavioral deficits in neurological patients using rating scales, which have acknowledged validity and reliability limitations [66]. Our results suggest that the reliability of clinical diagnostic procedures could be improved and simplified by using deep network scoring of patients’ movements. In principle, the approach presented here suggests that movement could be recorded using a phone camera and immediately scored by using an appropriate neural network application. This method would aid to standardize diagnosis and monitoring of neurological disorders and could be used by patients at home for daily symptoms monitoring.

## Methods

### Ethics statement

All animal experiments have been approved by the University of Lethbridge Animal Welfare Committee (protocol# 0907) in accordance with the Canadian Council on Animal Care.

### Deep neural network model

The general network architecture is shown in Fig 1. First, a deep convolutional neural network (ConvNet) is used to extract 2,048 high-level features from each frame. Here, we used a pretrained network called Inception-V3 [36]. Although this network was originally trained on the ImageNet dataset (images from 1,000 different classes like "dog," "car," "make-up," etc.), this network performed surprisingly well on our datasets (retraining top layers of this network specifically on our videos did not improve network performance). Next, features extracted by ConvNet from each video clip were combined (2,048 feature × 125 frames) and used as inputs to a RNN. This allowed the RNN to use sequences of movements to score neurological deficits. The RNN was composed of 2,048 long short-term memory (LSTM) units in the first layer, followed by maximum pooling, flatten and dropout layers, and then a single output neuron with the ReLU activation function that gave the movement disability score (S1 Table). Modifying the network architecture of our RNN and retraining it did not affect the results (S4 Fig). This shows that our network does not require fine parameter tuning to robustly converge to a good solution.

Code for our network and sample videos to reproduce our results are available at https://github.com/hardeepsryait/behaviour_net. The code was developed based on the Inception-V3 ConvNet [36] and based on code for recurrent network for identifying action classes in videos (https://github.com/harvitronix/five-video-classification-methods). A description of the main network parameters is provided in S1 Table and S4 Fig, and all the details of the network architecture, including all parameters, are available in our GitHub repository listed above.

### Knowledge extraction from neural network

Our network is composed of two separate networks: ConvNet and an RNN (Fig 1). To extract knowledge of feature attribution, we computed how much each ConvNet output feature (output unit) contributed to the RNN output (movement deficit score). Next, we found which parts of each frame were contributing to the activity of the most informative ConvNet output units. For this knowledge extraction from the ConvNet and RNN, we used the layer-wise relevance propagation method [38]. This method uses the strength of synaptic weights and neuronal activity in the previous layer to recursively calculate the contribution (relevance) of each neuron to the output score. For this, we used the DeepExplain package, available at github.com/marcoancona/DeepExplain, which is described elsewhere [39]. Using different knowledge extraction methods like saliency maps [67] gave qualitatively similar results.

### SPRT

Analyses of skilled forelimb reaching were performed as described in [68,69]. Each session required the rats to reach for a food pellet 20 consecutive times. Baseline training was considered complete once the success rate (pellet brought to mouth) reached asymptotic levels. After all animals were considered well trained, reaching trials were video recorded from a frontal view. Seven reaching movement components were scored according to earlier descriptions [3,69], based on procedures developed from the Eshkol-Wachman movement analysis [26]: (1) Limb lift: The reaching hand is initially located on the floor and supports body weight, with the fingers open and extended. As the arm is lifted using movements around the shoulder, the fingers first close and then flex, and the tips of the fingers align with the midline of the body. (2) Aim: With a movement at the shoulder, the elbow is adducted until the elbow and the fingertips are both oriented along the longitudinal midline axis of the body. For the hand to remain on the body midline, the movement involves concurrent abduction of the hand. The dual movement, elbow-adduct and hand-abduct, is termed a fixation because the hand remains at a fixed body-wise location. (3) Advance: The hand is advanced forward through the slot using a movement around the shoulder and may also be associated with forward body movement. During advance, the fingers extend. (4) Pronation: As the limb reaches maximum outward movement, the hand is pronated by abduction at the elbow and rotation at the wrist. During pronation, the fingers open in the sequence finger 5 through to finger 2 in an arpeggio-like movement. (5) Grasp: As the hand pronates, the fingers contact the food, following which they flex and close around the food target. As the grasp is completed, the food is lifted by a slight extension at the wrist. The grasp takes place with the hand in place. (6) Supination I: As the hand holding the food is withdrawn by movement at the shoulder and a backward postural shift, the hand is supinated by about 90°, mainly by adduction of the elbow. (7) Supination II: As the hand is brought toward the mouth, the hand supinates by a further <90° so that the palm of the hand is oriented toward the mouth.

Movement elements are rated using a three-point scale: 0, 0.5, or 1. A normal movement is given “0” score, an abnormal movement a score of “0.5,” and an absent movement a score of “1.” The movement ratings are made by stepping through the video record frame-by-frame and applying a score to each movement. For each reaching trial, a movement disability is calculated for each movement, and a sum of scores gives an overall assessment of severity.

Sixteen male Long-Evans rats, 3 months old and weighing 300–400 g, were used in this experiment, out of which nine rats had induced lesions in the forelimb area of primary motor cortex by a focal photothrombosis method [70,71]. To induce photothrombotic lesions, the skull over the motor cortex on the side contralateral to the skilled forelimb for reaching was thinned using a fine dental burr in a rectangular shape from −1.0 to 4.0 mm (posterior–anterior) to bregma and −1.0 to 4.0 mm lateral to the midline. A cold light source (Schott KL 1500, Germany) with an aperture of the same size and shape as the partial craniotomy was positioned over the skull. The skull was illuminated at maximum light settings for 20 minutes. During the first 2 minutes of illumination, Bengal Rose dye solution was injected through a tail vein (20 mg/kg, 10% solution in 0.9% saline; for control rats, only saline was injected).

The videos were recorded over 5 days: a day before surgery and on days 1, 6, 11, and 15 after surgery. From 16 rats, we used 692 video clips, in which each single clip corresponds to a single reaching trial. We used a Panasonic camera (HDC-TM900) at 60 frames per second. To train the neural network, we reduced the size of each frame to 300 × 300 pixels, centered roughly around the initial location of food pellet, which was held in a small indentation (Fig 1). To reduce training time, videos were also downsampled to 30 frames per second and divided in 125-frame-long clips, each consisting of a single reaching trial. Neither using the original sampling rate nor extending the clip length improved network performance.

### Stroke analysis

Nissl-stained coronal sections (40 μm thick), cut on a freezing microtome and mounted on microscope slides, were digitally scanned at 40× magnification (Nanozoomer, Hamamatsu Photonics, Hamamatsu, Japan). The images were transferred to the ImageJ software (NIH, Bethesda, MD, USA), and the lesion volumes were quantified. Volumes were measured by tracing the lesion borders and then multiplying the lesion area by section thickness and number of sections in the series.

### Parallel-beam-walking task

The parallel-beam task (PBT), in which animals walk across two elevated parallel beams, is commonly used to assess motor deficits in laboratory rodents. Twenty-one male Long-Evans rats, 3 months old and weighing 260–330 g, were used. Eleven rats received motor cortex devascularization lesions as described previously [17,72]. Rats were anesthetized, and the skin over the skull was opened and trephined on the side contralateral to the skilled forelimb for reaching using a fine dental burr. The coordinates for the maximum size trephination was 1 to 4.5 mm lateral to the midline and −1 to 4 mm anterior to bregma (caudal and rostral forelimb area of motor cortex). Within this area, the dura was carefully removed using microscissors. The underlying tissue was devascularized by wiping the pia and blood vessels away with a cotton tip. The skin was sutured after devascularization.

The PBT requires accurate interlimb coordination and balance while left and right limbs are maneuvered along two distant parallel beams. The task apparatus was elevated 30 cm above the ground with a refuge (home cage) at the end. A mirror, spanning the length of the walking platform, was positioned under the walking platform at a 45° angle, which provided a ventral view of the animals. All animals were habituated to the task and trained to traverse the PBT from the neutral start platform to reach their refuge (home cage) at the other end. Scoring systems for the PBT are described in detail in [17]. Briefly, three movement features were scored: (1) deviation from the correct placement of the paw on the beam surface, (2) time to complete the walk, and (3) average number of placing attempts per step. For the network training, each of those measures was normalized between 0 and 1, and an average across all three measures was used as a motor-disability score. In all experiments, we used “leave-one-animal-out” cross-validation, in which all videos from the predicted animal were excluded from the training dataset.

### Cross-validation

In all presented analyses, we used “leave-one-rat-out” cross-validation. That is, we trained the network on videos of 15 out of 16 rats from all 5 days and predicted expert scores for one remaining rat for all days. Thus, all analyses were repeated 16 times, and reported results are always from rats excluded from the training dataset. The same cross-validation procedure was applied to predictions using a linear model and using DeepLabCut features. The one exception to this procedure was a control experiment in which we used “leave-1-day-out” cross-validation to test how well the network can generalize to data recorded on different days (i.e., slightly different video recording conditions). In that case, we used videos from all rats from 4 days, and we predicted expert scores for all rates on the remaining day. This procedure was repeated five times for each excluded day separately. Considering that all our networks (trained to predict expert scores, stroke versus control, stroke size, movement element impairments) converged to similar solutions (see section above Fig 9) provides additional argument for the robustness of our approach. Matlab code and data used for the presented analyses and figures are available upon request from AL.

## Supporting information

S1 Fig Network scoring was within the human scoring variability range. Each point represents the average movement score for a single animal. Colors depict scores made by different researchers (yellow, green, red) and by the network (blue). Solid lines show linear regression for each researcher score and for the network. Identity line is shown as dashed. For each rat, we measured the absolute value of difference between the expert and other researcher scores. To quantify whether network performance was statistically different from that of the trained researchers, we used paired *t* tests to compare the distributions of differences; i.e., |expert score − researcher#*i* scores| versus |expert score − network scores|, where |…| denotes absolute value, and *i* is 1, 2, or 3. For all researchers, *p* > 0.1 (*p*_1_ = 0.27, *p*_2_ = 0.13, *p*_3_ = 0.92), showing that the network scores were not statistically distinguishable from researchers in reproducing expert scores.
(TIF)

S2 Fig Histogram of distance between normalized scores and stroke volume. Distribution of network scores closer to zero shows that network scores were better correlated with stroke volume than were expert scores (Wilcoxon signed rank test *p =* 0.0013).
(TIF)

S3 Fig The correlation between network scores and expert scoring across days for individual rats. Each dot represents a score for a single rat on a single day. Different colors represent different rats. Solid lines represent the regression for each rat. Distribution of regression lines along an identity line (dashed) shows that the network can predict changes in rat performance across days. Insert shows the distribution of correlation coefficients between network and expert scores for each rat (mean r = 0.67). Strong skewness of the distribution to the right shows that for the majority of rats, the network very accurately traced individual changes across days.
(TIF)

S4 Fig Robustness of the network performance with respect to changes in its architecture. Blue, red, and yellow dots represent network scores for model 1, 2, and 3, respectively (parameters of each model are listed in S1 Table below). Distribution of points along identity line (dashed) shows that all networks converged to a similar solution. Rationale for model parameter selection: Our network was composed of two parts—the convolutional network part (Inception V3) to extract features from frames and a recurrent network to combine information from multiple frames to make predictions about movement impairments (Fig 1). The convolutional network was previously optimized to extract features from images [36]. Retraining the last two blocks (i.e., freezing the first 249 layers and unfreezing the rest) of the convolutional network on our videos did not improve the accuracy of predictions. This suggests that the original Inception V3 network extracts image features useful for subsequent stroke disability predictions. Based on this, we used the original parameters for the convolutional network part. For the recurrent network, we made three significant modifications to layer structure and to the number of neurons (S1 Table). Consistency of results across such significantly modified recurrent networks suggests that our results are robust to network changes; thus, we did not test any further modifications.
(TIF)

S5 Fig The network can be trained to predict deficits in individual movement elements. (A) Each dot represents the score for a single movement element for a single rat averaged over all trials. Colors represent different movement elements: lift, aim, advance, pronation, grasp, supination I (“Sup1”), and supination II (“Sup2”) (see insert for color legend). The line represents least-squares regression fitted to all points regardless of movement element group. For those predictions, we modified our RNN to have output neurons corresponding to each movement component. Thus, a single network was trained to score all individual movement components. (B) Predicting stroke lesion volume from individual movement elements. A simple sum of scores from all 7 movement elements may not be the optimal predictor of stroke volume. For that, we used least-squares regression, which appropriately weighted each movement score to best predict stroke size. We applied this method to individual movements’ scores provided by the expert (yellow points) and separately to movement element scores predicted by the network (blue points). To prevent model overfitting, we used leave-one-rat-out cross-validation as described in the Methods. Results in this figure also show that when the network is trained to reproduce scores of individual movement elements, its stroke volume predictions are more similar to human scoring (compare to Fig 2C). This suggests that the network in Fig 2C learned to use temporal combinations of movement features to predict stroke severity. When predicting severity from individual movement elements, the absence of information about temporal relations reduces predictability. (C) Correlation coefficient between individual movement scores (calculated from expert scores) shows that movement components closer in time tend to have higher correlation of impairment scores. We found that out of 7 movement elements, the lift component had the highest correlation with stroke volume (R_Lift_ = 0.3592; R_Aim_ = 0.2879; R_Adv_ = 0.2446; R_Pron_ = 0.2651; R_Grasp_ = 0.2889; R_Sup1_ = −0.0005; R_Sup2_ = −0.0846). Supination had the lowest correlation with stroke volume; however, this should be taken with caution because in our dataset, two control rats had poor supination scores (see “Single movement element analyses” section). In Fig 3B and 3C, we show that in video clips, the network could discriminate individual movement components: (1) lift, (2) aim and advance, (3) pronation, (4) grasp, and (5) supination. To quantify network performance on this multiclass classification task, we also calculated classification accuracy, precision, and recall as described in [73]. Because the videos were divided in consecutive segments of seven frames, each segment could have transitions between movement elements, or more than one movement element within a segment. We considered the network prediction a success if it was within ±1 movement element category, as compared to human evaluator classification. The average network accuracy was 80.2%, precision was 77.7%, and recall was 83.4%. Using the same methods to compare classification made by two human evaluators gave similar results: average accuracy was 86.5%, precision was 83.8%, and recall was 83.9%. This again shows that the network can classify movement elements in video clips in a way similar to human evaluators.
(TIF)

S6 Fig The same outline of subclusters corresponding to individual movement components as shown in Fig 5, but with added examples of frames from subclusters. We selected frames from the closest to the subcluster center. Each of three frames shown for a single subcluster is selected from a different rat. Note that frames at the bottom show examples of successful and unsuccessful grasps. Rationale for dividing data in 40 subclusters: The typical number of movement elements defined by experts in reaching task is between 7 and 10. Each movement element can significantly differ between stroke and control animals; for example, the pronation cluster may be divided in two or more distinct subclusters corresponding to level of impairment (Fig 5). Moreover, at the beginning and at the end of a trial, there could be other movements—e.g., rearing or walking—which likely would form separate clusters in feature space. Therefore, to be able to differentiate all those possibly distinct movement subclusters, we decided to divide the data into 40 clusters. The fit of an ellipse to a given set of points was done by minimizing the least-squares criterion. Ellipses were only used for data visualization and were not used for data analyses.
(TIF)

S7 Fig Changes in movement element probability during stroke recovery. Each dot represents a subcluster corresponding to single movement component. y-Axis (M1) is a ratio of movement probability on day 1 after stroke and prestroke, and x-axis (M6) is a ratio of movement probabilities on days 6–15 after stroke in relation to prestroke probability. Specifically, here we investigated changes during the poststroke period across all subclusters (movement components). For this, within each subcluster *j*, we counted the number of points from the control period (p0_j_), the number of points from day 1 after stroke (p1_j_), and the sum of points from days 6, 11, and 15 after stroke (p6_j_) (those counts were then converted to probabilities by dividing by the total number of points on a given day). To see the changes in relation to the prestroke period for each subcluster, we calculated measures: M1_j_ = p1_j_/p0_j_ and M6_j_ = p6_j_/p0_j_ (for numerical stability to avoid division by 0, we added a small epsilon = 0.0001 to all p_j_). In other words, M1_j_ is a ratio of red bar (day 1) to blue bar (day before stroke) in Fig 6Ba or 6Bb. Plotting M1 versus M6 showed that about the third of the movement components effectively disappeared after stroke, with a similar number of new movement elements emerging after stroke. The distributions of points above the diagonal for “novel” movements and below the diagonal for “lost” movements indicates improvements in movement during the recovery period (days 6–15). To estimate how “elongated” the distribution of points along diagonal is, we calculated the correlation coefficient between log(M1) and log(M6) values for each rat separately. We found that more elongated distributions correlated with higher values of movement disability scores (r = 0.58, *p* < 0.001). Those analyses illustrate that the internal network representation can express complex movements in a simple low-dimensional representation, which allows for a detailed tracking of stroke recovery.
(TIF)

S8 Fig Measuring similarity of movement trajectories. (A) Any point in a movement trajectory is defined by values in PCs’ coordinates. To compare two trajectories, we concatenated the values of PC components in a single vector, separately for each trial. (B) Next, we calculated the cross-correlogram between both vectors, and the maximum value of the cross-correlogram was used as a measure of similarity between two movement trajectories. (C) Using the cross-correlogram enables the detection of similar movements, even if one movement started at a different time in relation to the beginning of the video clip. We used the first 7 PCs to measure the similarity of trajectories. We have chosen 7 PCs based on examination of eigenvalues, but changing the number of PCs between 5 and 15 did not affect our conclusions. (D) To measure distance between trajectories in a manner robust to changes in movement velocity, we also applied dynamic time warping (https://www.mathworks.com/matlabcentral/fileexchange/43156-dynamic-time-warping-dtw). Using dynamic time warping gave similar results to that using cross-correlation measure (compare with Fig 7; note that in this plot, smaller values of distance indicate larger similarity of trajectories). PC, principal component.
(TIF)

S9 Fig Shifts in camera angle cause large changes to PCA space. (A) As a control experiment, we applied PCA to all video frames to use it as features. As a result, each video frame was represented by 20 principal components, accounting for most of the variance. Next, we applied a least-squares regression to predict the expert scores from PCA scores. This did not give a significant result (r = −0.1 *p* = 0.69). For selecting the number of PCA components, we use the scree plot method [74]. Specifically, in the above scree plot of eigenvalues, we selected a “knee” point at which values level off. This is a generally used rule of thumb, as there is no theoretically optimal method for selecting the number of PCA components. To ensure this does not introduce bias, we varied the number of selected PCA components between 10 and 100, which gave consistent results. (B) To investigate why our predictions from PCA scores failed, we visualized the first 20 principal components in 2D using t-SNE [42], a nonlinear dimensionality reduction technique. Each point corresponds to a single frame in the t-SNE projection. Data from each rat are marked with a different color. For clarity, only data from nine rats and 1 day are shown, when the camera was accidentally moved after filming rat #5. The camera shift caused large change in PCA features as evident by separate cluster for rats 6–9. The average frame for rats 1–5 and rats 6–9 is shown on the right side. (C) Using 2,048 principal components and the RNN improved the predictions toward expert scores. However, it was still markedly worse than using 2,048 ConvNet features and RNN (compare to Fig 2B). This is likely due to fact that convolutional networks with pooling layers, as used in our approach, can extract features which are robust to spatial shifts [75]. We also asked whether the dimensionality of the featurized image data changes before and after stroke. To test this, we did SVD on image features from ConvNet separately for data before and after stroke. We could not detect significant differences among the distributions of singular values. This suggests that dimensionality is likely too crude of a measure to detect movement differences induced by stroke. However, the amplitude of selected features allows for such discrimination, as described in Fig 4. ConvNet, convolutional network; PCA, principal component analysis; RNN, recurrent neural network; SVD, singular-value decomposition; t-SNE, t-distributed stochastic neighbor embedding.
(TIF)

S10 Fig (A) Sample frames with marked body parts by DeepLabCut. (B) We used the RNN to predict expert scores from coordinates of points marked by DeepLabCut and from its confidence levels. The correlation coefficient between predicted and actual expert scores was r = 0.53, *p* = 0.036. All x- and y-coordinates were divided by 300 to be in the 0–1 range. For points assigned to body parts that were not visible in a frame or were difficult to identify, DeepLabCut gave a confidence level close to 0. Adding 4 more markers on body parts did not significantly change predictions. We also tried other software to track body parts: LEAP [43], which gave comparable results to DeepLabCut. RNN, recurrent neural network.
(TIF)

S11 Fig Network validation on the mouse string-pulling task. (Left) Sample frame of a mouse during the string-pulling task. For this task, we trained the network to discriminate control from stroke mice. (Right) Network scores of movement deficits. Bars show group average, and crosses show scores for individual mice. The network learned to discriminate between both groups with 100% accuracy (note: no overlap in scores between groups). String-pulling task: This task examines the coordination of bilateral hand and arm movements used in spontaneous string pulling. Procedures for training mice in the string-pulling task were based on an earlier description of the behavior [5]. Briefly, 11 Chat-CreAi32 mice (5 females, 6 males) that were 3–5 months old, weighed 20–30 g, and were raised at the Canadian Centre for Behavioural Neuroscience Vivarium at the University of Lethbridge were weighed and placed on food restriction 3 days prior to training. Food reinforcement on the end of the string was used as motivation for string pulling. Mice were weighed daily to maintain body weight at 90% of pre-restriction weight. They were given additional food in their home cage 2 hours after completion of daily training/testing. On each training/testing day, animals were individually placed in a clear plastic container for transport to the testing room. Mice were trained for 3 days to pull a 90-cm-long piece of string hanging from the top of a transparent Plexiglas box to obtain the food reward tied at the end, and then they were filmed. They underwent photothrombotic stroke induction in their primary forelimb somatosensory area and were filmed in the string-pulling task before stroke and on day 4 poststroke. Animals were kept on food restriction throughout the experiment. No scoring system has been developed for this task; thus, we only trained network to classify stroke versus control condition with pre- and poststroke videos.
(TIF)

S1 Table RNN parameters. Numbers in brackets show the number of neurons in a layer. LSTM neurons can remember their previous state, which makes them particularly useful for analyzing temporal sequences, as used here for scoring movements in video clips. The max-pooling layer down-samples an input representation, which reduces computational complexity and helps to find shift-invariant features. We also used a dropout layer with value 0.1, meaning that at each time, 10% of randomly selected neurons were set to 0. This procedure helps the network to avoid overfitting. Dense layer refers to output neurons that receive inputs from all neurons in the layer below. For our RNN, we used ADAM optimizer with 10^−6^ learning rate and with “mean squared error” loss function. Our batch size was set to 8, and changing it to 16 did not improve predictions. We set number of training epochs to 1,000, with steps per epoch = number of training data/batch size. From 16 rats, we had 692 recorded trials, with mean number of trials per rat = 43.25 ± 5.2 SEM (because of variability in rat performance, not all rats did the same number of trials). We used a cross-validation method with 16 replications. This means that during each training session, data from one rat were excluded (Methods); thus, about 649 video clips were used for training and about 43 for testing. To predict expert scores, the network was trained for regression task. LSTM, long short-term memory; RNN, recurrent neural network.
(XLSX)

## Abbreviations

a.u.: arbitrary units
ConvNet: convolutional network
eLRP: ϵ-layer-wise relevance propagation
LSTM: long short-term memory
Movement comp. prob.: movement component probability
PC: principal component
PCA: principal component analysis
RNN: recurrent neural network
SPRT: single-pellet reaching task
t-SNE: t-distributed stochastic neighbor embedding

## References

[1] Krakauer JW, Ghazanfar AA, Gomez-Marin A, MacIver MA, Poeppel D. Neuroscience needs behavior: correcting a reductionist bias. Neuron. 2017;93(3):480–90. 10.1016/j.neuron.2016.12.041 28182904

[2] Mench J. Why it is important to understand animal behavior. ILAR journal. 1998;39(1):20–6. 10.1093/ilar.39.1.20 11528062

[3] Alaverdashvili M, Whishaw IQ. A behavioral method for identifying recovery and compensation: hand use in a preclinical stroke model using the single pellet reaching task. Neuroscience & Biobehavioral Reviews. 2013;37(5):950–67. 23583614 10.1016/j.neubiorev.2013.03.026

[4] Karthikeyan S, Jeffers MS, Carter A, Corbett D. Characterizing Spontaneous Motor Recovery Following Cortical and Subcortical Stroke in the Rat. Neurorehabilitation and neural repair. 2018:1545968318817823. 10.1177/1545968318817823 30526316

[5] Blackwell AA, Banovetz MT, Qandeel, Whishaw IQ, Wallace DG. The structure of arm and hand movements in a spontaneous and food rewarded on-line string-pulling task by the mouse. Behav Brain Res. 2018;345:49–58. Epub 2018/02/24. 10.1016/j.bbr.2018.02.025 . 29474809

[6] Klein A, Sacrey L-AR, Whishaw IQ, Dunnett SB. The use of rodent skilled reaching as a translational model for investigating brain damage and disease. Neuroscience & Biobehavioral Reviews. 2012;36(3):1030–42. 22227413 10.1016/j.neubiorev.2011.12.010

[7] Nielson JL, Paquette J, Liu AW, Guandique CF, Tovar CA, Inoue T, et al Topological data analysis for discovery in preclinical spinal cord injury and traumatic brain injury. Nature communications. 2015;6:8581 10.1038/ncomms9581 26466022 PMC4634208

[8] Rattka M, Fluri F, Krstić M, Asan E, Volkmann J. A Novel Approach to Assess Motor Outcome of Deep Brain Stimulation Effects in the Hemiparkinsonian Rat: Staircase and Cylinder Test. Journal of visualized experiments: JoVE. 2016;(111). 10.3791/53951 PMC4927743 27284739

[9] Sacrey L-AR, Alaverdashvili M, Whishaw IQ. Similar hand shaping in reaching-for-food (skilled reaching) in rats and humans provides evidence of homology in release, collection, and manipulation movements. Behavioural brain research. 2009;204(1):153–61. 10.1016/j.bbr.2009.05.035 19520119

[10] Brown AR, Teskey GC. Motor cortex is functionally organized as a set of spatially distinct representations for complex movements. Journal of Neuroscience. 2014;34(41):13574–85. 10.1523/JNEUROSCI.2500-14.2014 25297087 PMC6608383

[11] Jones TA. Motor compensation and its effects on neural reorganization after stroke. Nature Reviews Neuroscience. 2017;18(5):267 10.1038/nrn.2017.26 28331232 PMC6289262

[12] Metz G, Dietz V, Schwab M, Van de Meent H. The effects of unilateral pyramidal tract section on hindlimb motor performance in the rat. Behavioural brain research. 1998;96(1–2):37–46. 10.1016/s0166-4328(97)00195-2 9821541

[13] Metz GA, Schwab ME, Welzl H. The effects of acute and chronic stress on motor and sensory performance in male Lewis rats. Physiology & behavior. 2001;72(1–2):29–35. 11239978 10.1016/s0031-9384(00)00371-1

[14] Metz GA, Whishaw IQ. Cortical and subcortical lesions impair skilled walking in the ladder rung walking test: a new task to evaluate fore-and hindlimb stepping, placing, and co-ordination. Journal of neuroscience methods. 2002;115(2):169–79. 10.1016/s0165-0270(02)00012-2 11992668

[15] Metz GA, Whishaw IQ. The ladder rung walking task: a scoring system and its practical application. Journal of visualized experiments: JoVE. 2009;(28). 10.3791/1204 PMC2796662 19525918

[16] Faraji J, Kurio K, Metz GA. Concurrent silent strokes impair motor function by limiting behavioral compensation. Experimental neurology. 2012;236(2):241–8. 10.1016/j.expneurol.2012.05.007 22609330

[17] Ficiur B, Faraji J, Metz GA. Use of the parallel beam task for skilled walking in a rat model of cerebral ischemia: A qualitative approach. Learning and Motivation. 2018;61:74–84.

[18] Carmona C, Wilkins KB, Drogos J, Sullivan JE, Dewald J, Yao J. Improving Hand function of Severely Impaired Chronic Hemiparetic Stroke Individuals using Task Specific Training with the ReIn-Hand system: A Case Series. Frontiers in neurology. 2018;9:923 10.3389/fneur.2018.00923 30464754 PMC6234834

[19] Foroud A, Whishaw IQ. Changes in the kinematic structure and non-kinematic features of movements during skilled reaching after stroke: A laban movement analysis in two case studies. Journal of neuroscience methods. 2006;158(1):137–49. 10.1016/j.jneumeth.2006.05.007 16766042

[20] Butt A, Rovini E, Dolciotti C, De Petris G, Bongioanni P, Carboncini M, et al Objective and automatic classification of Parkinson disease with Leap Motion controller. Biomedical engineering online. 2018;17(1):168 10.1186/s12938-018-0600-7 30419916 PMC6233603

[21] Doan JB, Melvin KG, Whishaw IQ, Suchowersky O. Bilateral impairments of skilled reach-to-eat in early Parkinson's disease patients presenting with unilateral or asymmetrical symptoms. Behavioural brain research. 2008;194(2):207–13. 10.1016/j.bbr.2008.07.015 18692094

[22] Whishaw IQ, Suchowersky O, Davis L, Sarna J, Metz GA, Pellis SM. Impairment of pronation, supination, and body co-ordination in reach-to-grasp tasks in human Parkinson's disease (PD) reveals homology to deficits in animal models. Behavioural brain research. 2002;133(2):165–76. 10.1016/s0166-4328(01)00479-x 12110450

[23] Klein A, Sacrey L-AR, Dunnett SB, Whishaw IQ, Nikkhah G. Proximal movements compensate for distal forelimb movement impairments in a reach-to-eat task in Huntington's disease: new insights into motor impairments in a real-world skill. Neurobiology of disease. 2011;41(2):560–9. 10.1016/j.nbd.2010.11.002 21059390

[24] Kleinman S. Movement notation systems: An introduction. Quest. 1975;23(1):33–4.

[25] Teitelbaum P, Teitelbaum O, Nye J, Fryman J, Maurer RG. Movement analysis in infancy may be useful for early diagnosis of autism. Proceedings of the National Academy of Sciences. 1998;95(23):13982–7. 10.1073/pnas.95.23.13982 PMC25000 9811912

[26] Whishaw IQ, Pellis SM. The structure of skilled forelimb reaching in the rat: a proximally driven movement with a single distal rotatory component. Behav Brain Res. 1990;41(1):49–59. Epub 1990/12/07. doi: 0166-4328(90)90053-H [pii]. 10.1016/0166-4328(90)90053-h . 2073355

[27] Cenci MA, Whishaw IQ, Schallert T. Animal models of neurological deficits: how relevant is the rat? Nature Reviews Neuroscience. 2002;3(7):574 10.1038/nrn877 12094213

[28] Hylin MJ, Kerr AL, Holden R. Understanding the mechanisms of recovery and/or compensation following injury. Neural plasticity. 2017;2017. 10.1155/2017/7125057 PMC5415868 28512585

[29] Krizhevsky A, Sutskever I, Hinton GE, editors. Imagenet classification with deep convolutional neural networks In: Advances in neural information processing systems. Lake Tahoe, NV: NIPS; 2012 p. 1097–1105.

[30] Farabet C, Couprie C, Najman L, LeCun Y. Learning hierarchical features for scene labeling. IEEE transactions on pattern analysis and machine intelligence. 2013;35(8):1915–29. 10.1109/TPAMI.2012.231 23787344

[31] Taigman Y, Yang M, Ranzato MA, Wolf L. Deepface: Closing the gap to human-level performance in face verification In: Proceedings of the IEEE conference on computer vision and pattern recognition. Columbus, OH: IEEE Press; 2014 pp 1791–1708.

[32] He K, Zhang X, Ren S, Sun J. Delving deep into rectifiers: Surpassing human-level performance on imagenet classification In: Proceedings of the IEEE international conference on computer vision. Santiago, Chile: IEEE Press; 2015 pp: 1026–1034.

[33] Toshev A, Szegedy C. Deeppose: Human pose estimation via deep neural networks In: Proceedings of the IEEE conference on computer vision and pattern recognition. Columbus, OH: IEEE Press; 2014 pp 1653–1660.

[34] Tompson JJ, Jain A, LeCun Y, Bregler C. Joint training of a convolutional network and a graphical model for human pose estimation In: Advances in neural information processing systems. Montreal: MIT Press Cambridge; 2014 pp 1799–1807.

[35] Baccouche M, Mamalet F, Wolf C, Garcia C, Baskurt A. Sequential deep learning for human action recognition In: International Workshop on Human Behavior Understanding. Amsterdam: Springer-Verlag, Berlin; 2011 pp: 29–39.

[36] Szegedy C, Vanhoucke V, Ioffe S, Shlens J, Wojna Z. Rethinking the inception architecture for computer vision In: Proceedings of the IEEE conference on computer vision and pattern recognition. Las Vegas: IEEE Press; 2016 pp. 2818–2826

[37] Yamins DL, Hong H, Cadieu CF, Solomon EA, Seibert D, DiCarlo JJ. Performance-optimized hierarchical models predict neural responses in higher visual cortex. Proceedings of the National Academy of Sciences. 2014;111(23):8619–24. 10.1073/pnas.1403112111 PMC4060707 24812127

[38] Bach S, Binder A, Montavon G, Klauschen F, Müller K-R, Samek W. On pixel-wise explanations for non-linear classifier decisions by layer-wise relevance propagation. PLoS ONE. 2015;10(7):e0130140 10.1371/journal.pone.0130140 26161953 PMC4498753

[39] Ancona M, Ceolini E, Oztireli C, Gross M, editors. Towards better understanding of gradient-based attribution methods for Deep Neural Networks In: 6th International Conference on Learning Representations (ICLR 2018) 2018.

[40] Hurd C, Weishaupt N, Fouad K. Anatomical correlates of recovery in single pellet reaching in spinal cord injured rats. Experimental neurology. 2013;247:605–14. 10.1016/j.expneurol.2013.02.013 23470552

[41] Whishaw IQ. Loss of the innate cortical engram for action patterns used in skilled reaching and the development of behavioral compensation following motor cortex lesions in the rat. Neuropharmacology. 2000;39(5):788–805. 10.1016/s0028-3908(99)00259-2 10699445

[42] Lvd Maaten, Hinton G. Visualizing data using t-SNE. Journal of machine learning research. 2008;9(Nov):2579–605.

[43] Pereira TD, Aldarondo DE, Willmore L, Kislin M, Wang SS-H, Murthy M, et al Fast animal pose estimation using deep neural networks. Nature methods. 2019;16(1):117 10.1038/s41592-018-0234-5 30573820 PMC6899221

[44] Mathis A, Mamidanna P, Cury KM, Abe T, Murthy VN, Mathis MW, et al DeepLabCut: markerless pose estimation of user-defined body parts with deep learning Nature Publishing Group, 2018 1546–1726. 10.1038/s41593-018-0209-y 30127430

[45] Wold S, Sjöström M, Eriksson L. PLS-regression: a basic tool of chemometrics. Chemometrics and intelligent laboratory systems. 2001;58(2):109–30.

[46] McKenna JE, Whishaw IQ. Complete compensation in skilled reaching success with associated impairments in limb synergies, after dorsal column lesion in the rat. Journal of Neuroscience. 1999;19(5):1885–94. 10.1523/JNEUROSCI.19-05-01885.1999 10024372 PMC6782168

[47] Morris R, Whishaw IQ. A proposal for a rat model of spinal cord injury featuring the rubrospinal tract and its contributions to locomotion and skilled hand movement. Frontiers in neuroscience. 2016;10:5 10.3389/fnins.2016.00005 26858587 PMC4728831

[48] Burke JF, Yue JK, Ngwenya LB, Winkler EA, Talbott JF, Pan JZ, et al Ultra-Early (< 12 Hours) Surgery Correlates With Higher Rate of American Spinal Injury Association Impairment Scale Conversion After Cervical Spinal Cord Injury. Neurosurgery. 2019;85(2):199–203. 10.1093/neuros/nyy537 30496474

[49] Cenci MA, Jörntell H, Petersson P. On the neuronal circuitry mediating l-DOPA-induced dyskinesia. Journal of neural transmission. 2018;125:1157–69. 10.1007/s00702-018-1886-0 29704061 PMC6060876

[50] Torres-Espín A, Beaudry E, Fenrich K, Fouad K. Rehabilitative Training in Animal Models of Spinal Cord Injury. Journal of neurotrauma. 2018;35(16):1970–85. 10.1089/neu.2018.5906 30074874

[51] Moran RW, Schneiders AG, Major KM, Sullivan SJ. How reliable are Functional Movement Screening scores? A systematic review of rater reliability. Br J Sports Med. 2016;50(9):527–36. 10.1136/bjsports-2015-094913 26316583

[52] Zhou F, Duh HB-L, Billinghurst M. Trends in augmented reality tracking, interaction and display: A review of ten years of ISMAR In: Proceedings of the 7th IEEE/ACM International Symposium on Mixed and Augmented Reality. IEEE Computer Society. Washington, DC: IEEE Press; 2008 pp: 193–202.

[53] Dell AI, Bender JA, Branson K, Couzin ID, de Polavieja GG, Noldus LP, et al Automated image-based tracking and its application in ecology. Trends in ecology & evolution. 2014;29(7):417–28. 24908439 10.1016/j.tree.2014.05.004

[54] Anderson DJ, Perona P. Toward a science of computational ethology. Neuron. 2014;84(1):18–31. 10.1016/j.neuron.2014.09.005 25277452

[55] Matsumoto J, Urakawa S, Takamura Y, Malcher-Lopes R, Hori E, Tomaz C, et al A 3D-video-based computerized analysis of social and sexual interactions in rats. PLoS ONE. 2013;8(10):e78460 10.1371/journal.pone.0078460 24205238 PMC3813688

[56] Dollár P, Welinder P, Perona P. Cascaded pose regression Computer Vision and Pattern Recognition (CVPR), 2010 IEEE Conference. San Francisco: IEEE Press; 2010 pp: 1078–105.

[57] Machado AS, Darmohray DM, Fayad J, Marques HG, Carey MR. A quantitative framework for whole-body coordination reveals specific deficits in freely walking ataxic mice. Elife. 2015;4:e07892 10.7554/eLife.07892 26433022 PMC4630674

[58] Drai D, Golani I. SEE: a tool for the visualization and analysis of rodent exploratory behavior. Neuroscience & Biobehavioral Reviews. 2001;25(5):409–26. 11566479 10.1016/s0149-7634(01)00022-7

[59] Sousa N, Almeida O, Wotjak C. A hitchhiker's guide to behavioral analysis in laboratory rodents. Genes, Brain and Behavior. 2006;5:5–24. 10.1111/j.1601-183X.2006.00228.x 16681797

[60] Gomez-Marin A, Partoune N, Stephens GJ, Louis M. Automated tracking of animal posture and movement during exploration and sensory orientation behaviors. PLoS ONE. 2012;7(8):e41642 10.1371/journal.pone.0041642 22912674 PMC3415430

[61] Ben-Shaul Y. OptiMouse: a comprehensive open source program for reliable detection and analysis of mouse body and nose positions. BMC biology. 2017;15(1):41 10.1186/s12915-017-0377-3 28506280 PMC5433172

[62] Berman GJ, Choi DM, Bialek W, Shaevitz JW. Mapping the stereotyped behaviour of freely moving fruit flies. Journal of The Royal Society Interface. 2014;11(99):20140672. 10.1098/rsif.2014.0672 PMC4233753 25142523

[63] Wiltschko AB, Johnson MJ, Iurilli G, Peterson RE, Katon JM, Pashkovski SL, et al Mapping sub-second structure in mouse behavior. Neuron. 2015;88(6):1121–35. 10.1016/j.neuron.2015.11.031 26687221 PMC4708087

[64] Arac A, Zhao P, Dobkin BH, Carmichael ST, Golshani P. DeepBehavior: A deep learning toolbox for automated analysis of animal and human behavior imaging data. Frontiers in systems neuroscience. 2019;13:20 10.3389/fnsys.2019.00020 31133826 PMC6513883

[65] Jhuang H, Garrote E, Yu X, Khilnani V, Poggio T, Steele AD, et al Automated home-cage behavioural phenotyping of mice. Nature communications. 2010;1:68 10.1038/ncomms1064 20842193

[66] Nicholl DJ, Appleton JP. Clinical neurology: why this still matters in the 21st century. J Neurol Neurosurg Psychiatry. 2015;86(2):229–33. 10.1136/jnnp-2013-306881 24879832 PMC4316836

[67] Simonyan K, Vedaldi A, Zisserman A. Deep inside convolutional networks: Visualising image classification models and saliency maps. arXiv:13126034 [Preprint]. 2014 [cited 2014 Apr 19]. Available from: https://arxiv.org/abs/1312.6034

[68] Faraji J, Gomez-Palacio-Schjetnan A, Luczak A, Metz GA. Beyond the silence: bilateral somatosensory stimulation enhances skilled movement quality and neural density in intact behaving rats. Behavioural brain research. 2013;253:78–89. 10.1016/j.bbr.2013.07.022 23871611

[69] Metz GA, Whishaw IQ. Skilled reaching an action pattern: stability in rat (Rattus norvegicus) grasping movements as a function of changing food pellet size. Behav Brain Res. 2000;116(2):111–22. Epub 2000/11/18. doi: S016643280000245X [pii]. 10.1016/s0166-4328(00)00245-x . 11080542

[70] Metz GA, Antonow-Schlorke I, Witte OW. Motor improvements after focal cortical ischemia in adult rats are mediated by compensatory mechanisms. Behav Brain Res. 2005;162(1):71–82. Epub 2005/06/01. 10.1016/j.bbr.2005.03.002 . 15922067

[71] Schjetnan AGP, Gidyk D, Metz GAS, Luczak A. Direct Current Stimulation Improves Limb Use After Stroke by Enhancing Inter-hemispheric Coherence. Acta Neurobiologiae Experimentalis. 2019;79:290–301. 31587021

[72] Zucchi FC, Matthies N-F, Badr N, Metz GA. Stress-induced glucocorticoid receptor activation determines functional recovery following ischemic stroke. Experimental & translational stroke medicine. 2010;2(1):18. 20858282 10.1186/2040-7378-2-18 PMC2954925

[73] Sokolova M, Lapalme G. A systematic analysis of performance measures for classification tasks. Information processing & management. 2009;45(4):427–37.

[74] Cattell RB. The scree test for the number of factors. Multivariate behavioral research. 1966;1(2):245–76. 10.1207/s15327906mbr0102_10 26828106

[75] LeCun Y, Bengio Y, Hinton G. Deep learning. Nature. 2015;521(7553):436 10.1038/nature14539 26017442

