# Correction: Data-driven analyses of motor impairments in animal models of neurological disorders

**DOI:** 10.1371/journal.pbio.3002521

**Published:** 2024-02-08

**Authors:** Hardeep Ryait, Edgar Bermudez-Contreras, Matthew Harvey, Jamshid Faraji, Behroo Mirza Agha, Andrea Gomez-Palacio Schjetnan, Aaron Gruber, Jon Doan, Majid Mohajerani, Gerlinde A. S. Metz, Ian Q. Whishaw, Artur Luczak

Animal protocol number 0907 in the Methods section is incorrect. The correct protocol numbers approved by the University of Lethbridge Animal Welfare Committee are: 1007, 1008 and 1404.
