# Correction: Correction: Data-driven analyses of motor impairments in animal models of neurological disorders

**DOI:** 10.1371/journal.pbio.3004030

**Published:** 2026-09-29

**Authors:** The PLOS Biology Staff

There is an error in the citation for author Behroo Mirza Agha in the correction published on February 8, 2024. The correct citation is: Ryait H, Bermudez-Contreras E, Harvey M, Faraji J, Mirza Agha B, Gomez-Palacio Schjetnan A, and colleagues (2019) Data-driven analyses of motor impairments in animal models of neurological disorders. PLOS Biology 17(11): e3000516. https://doi.org/10.1371/journal.pbio.3000516.

## References

[1] Ryait H, Bermudez-Contreras E, Harvey M, Faraji J, Mirza Agha B, Gomez-Palacio Schjetnan A, et al. Data-driven analyses of motor impairments in animal models of neurological disorders. PLoS Biol. 2019;17(11):e3000516. doi: 10.1371/journal.pbio.3000516 31751328 PMC6871764

[2] Ryait H, Bermudez-Contreras E, Harvey M, Faraji J, Agha BM, Schjetnan AG-P, et al. Correction: Data-driven analyses of motor impairments in animal models of neurological disorders. PLoS Biol. 2024;22(2):e3002521. doi: 10.1371/journal.pbio.3002521 38330553 PMC10853014

