# Correction: Correction: Correction: Data-driven analyses of motor impairments in animal models of neurological disorders

**DOI:** 10.1371/journal.pbio.3004090

**Published:** 2026-11-05

**Authors:** The PLOS Biology Staff

## Notice of Republication

This article was republished on October 13, 2026, to correct an error in the byline of the correction. An incorrect journal was listed as the author of the correction. The byline should read: The *PLOS Biology* Staff. The publisher apologizes for the errors.

## Supporting information

S1 File Originally published, uncorrected article. (PDF)

S2 File Republished, corrected article. (PDF)
