# Supplementary figures and images for: Data-driven analyses of motor impairments in animal models of neurological disorders
Source: PLoS Biol. 2019 Nov 21;17(11):e3000516. doi: 10.1371/journal.pbio.3000516 (PMC6871764; doi:10.1371/journal.pbio.3000516)

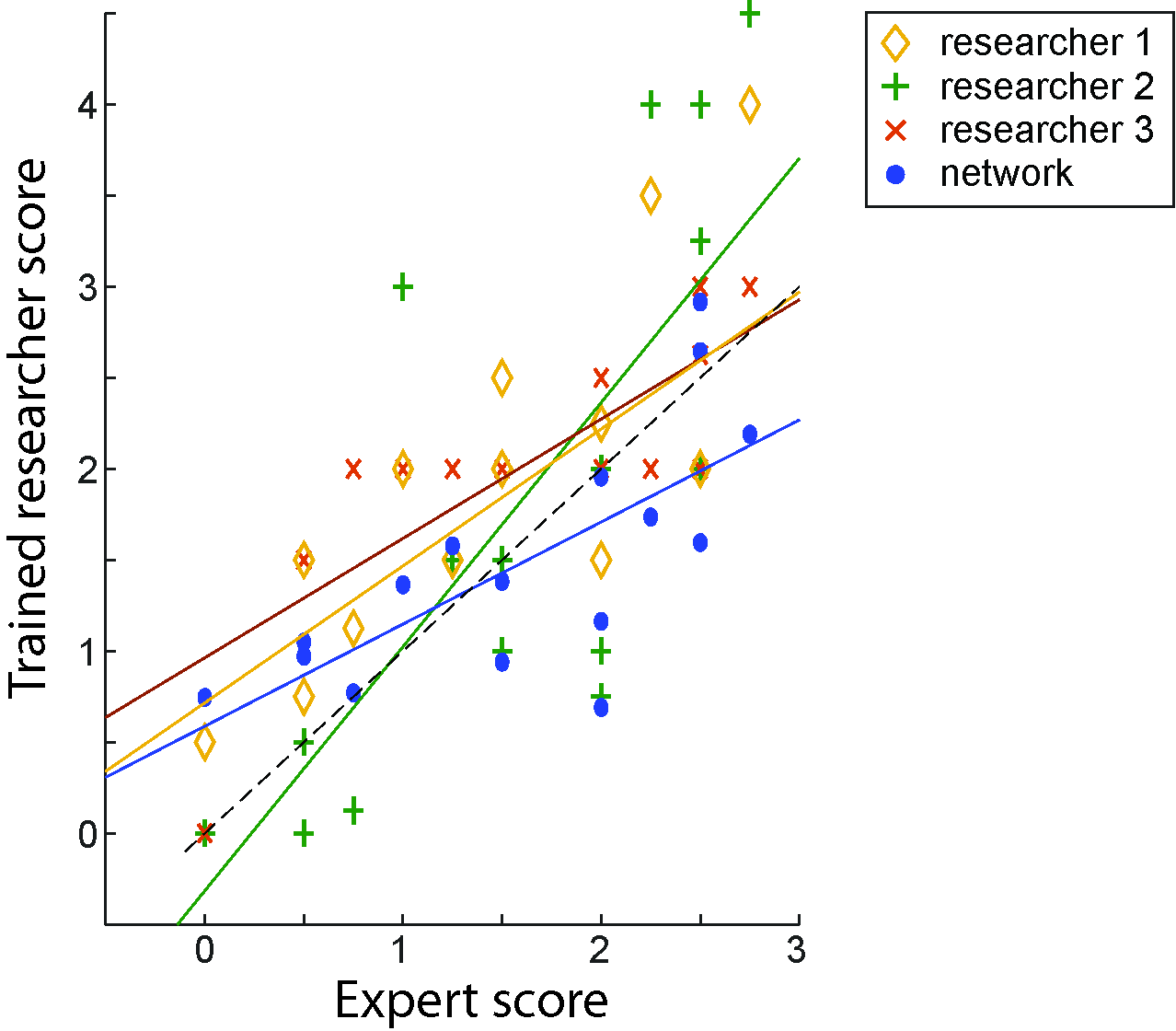

Supplement: S1 Fig — Each point represents the average movement score for a single animal. Colors depict scores made by different researchers (yellow, green, red) and by the network (blue). Solid lines show linear regression for each researcher score and for the network. Identity line is shown as dashed. For each rat, we measured the absolute value of difference between the expert and other researcher scores. To quantify whether network performance was statistically different from that of the trained researchers, we used paired t tests to compare the distributions of differences; i.e., |expert score − researcher#i scores| versus |expert score − network scores|, where |…| denotes absolute value, and i is 1, 2, or 3. For all researchers, p > 0.1 (p1 = 0.27, p2 = 0.13, p3 = 0.92), showing that the network scores were not statistically distinguishable from researchers in reproducing expert scores. (TIF) [file pbio.3000516.s001.tif]

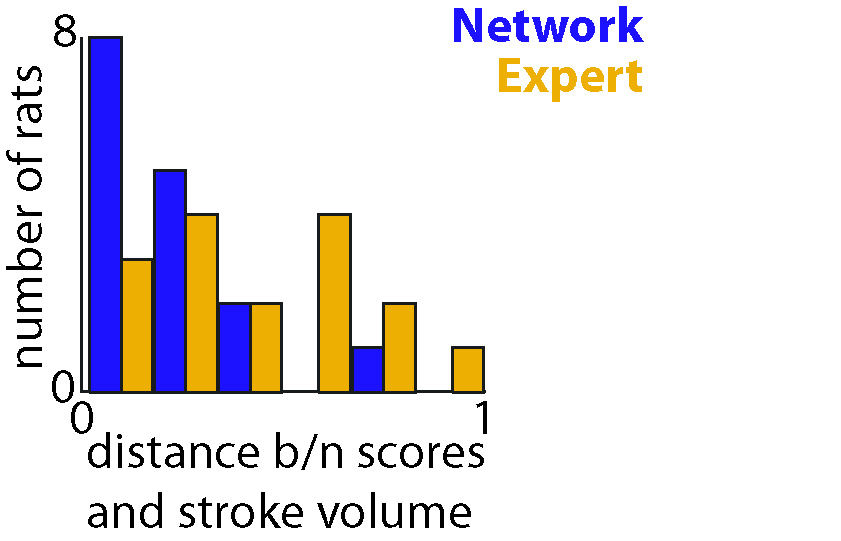

Supplement: S2 Fig — Distribution of network scores closer to zero shows that network scores were better correlated with stroke volume than were expert scores (Wilcoxon signed rank test p = 0.0013). (TIF) [file pbio.3000516.s002.tif]

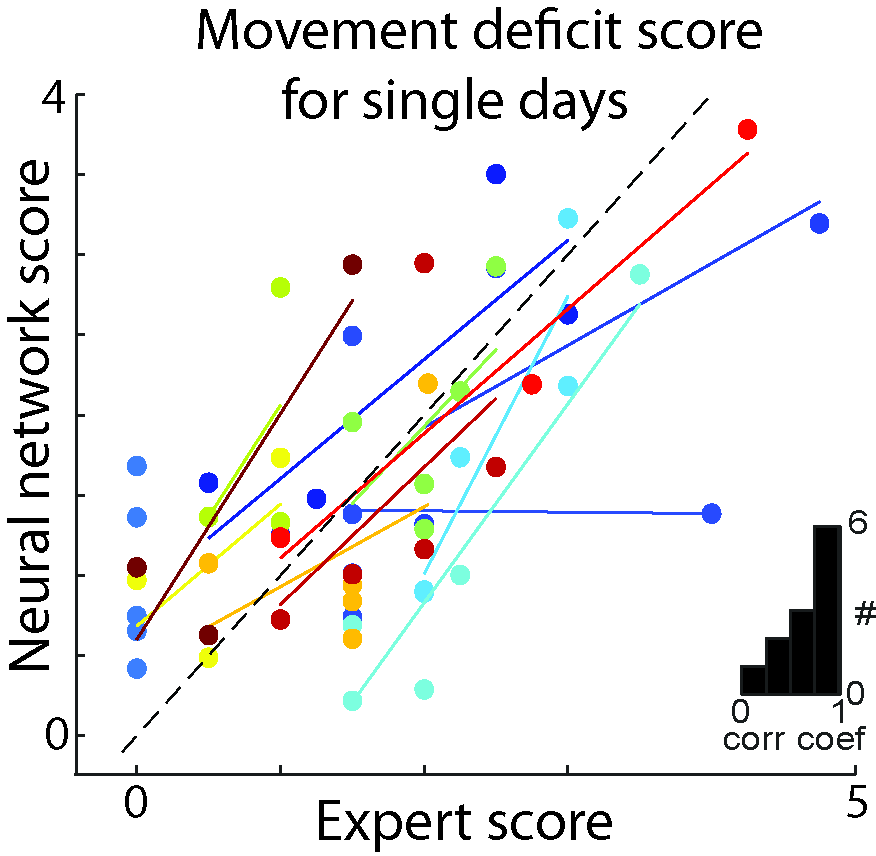

Supplement: S3 Fig — Each dot represents a score for a single rat on a single day. Different colors represent different rats. Solid lines represent the regression for each rat. Distribution of regression lines along an identity line (dashed) shows that the network can predict changes in rat performance across days. Insert shows the distribution of correlation coefficients between network and expert scores for each rat (mean r = 0.67). Strong skewness of the distribution to the right shows that for the majority of rats, the network very accurately traced individual changes across days. (TIF) [file pbio.3000516.s003.tif]

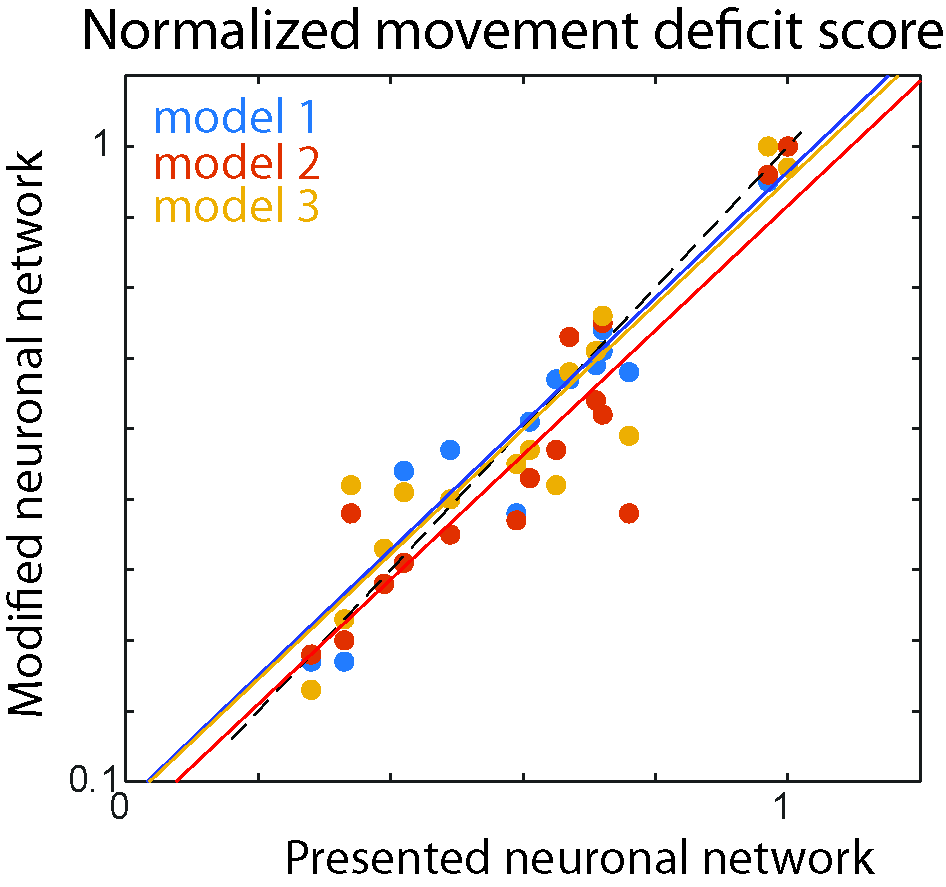

Supplement: S4 Fig — Blue, red, and yellow dots represent network scores for model 1, 2, and 3, respectively (parameters of each model are listed in S1 Table below). Distribution of points along identity line (dashed) shows that all networks converged to a similar solution. Rationale for model parameter selection: Our network was composed of two parts—the convolutional network part (Inception V3) to extract features from frames and a recurrent network to combine information from multiple frames to make predictions about movement impairments (Fig 1). The convolutional network was previously optimized to extract features from images [36]. Retraining the last two blocks (i.e., freezing the first 249 layers and unfreezing the rest) of the convolutional network on our videos did not improve the accuracy of predictions. This suggests that the original Inception V3 network extracts image features useful for subsequent stroke disability predictions. Based on this, we used the original parameters for the convolutional network part. For the recurrent network, we made three significant modifications to layer structure and to the number of neurons (S1 Table). Consistency of results across such significantly modified recurrent networks suggests that our results are robust to network changes; thus, we did not test any further modifications. (TIF) [file pbio.3000516.s004.tif]

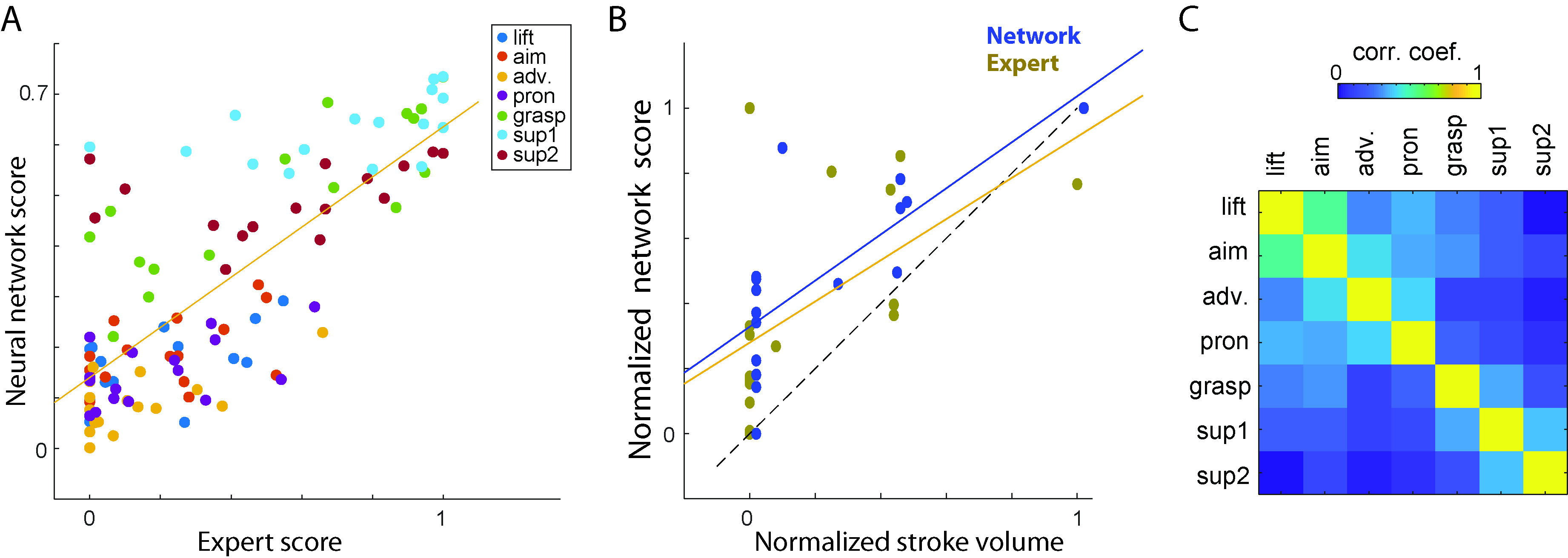

Supplement: S5 Fig — (A) Each dot represents the score for a single movement element for a single rat averaged over all trials. Colors represent different movement elements: lift, aim, advance, pronation, grasp, supination I (“Sup1”), and supination II (“Sup2”) (see insert for color legend). The line represents least-squares regression fitted to all points regardless of movement element group. For those predictions, we modified our RNN to have output neurons corresponding to each movement component. Thus, a single network was trained to score all individual movement components. (B) Predicting stroke lesion volume from individual movement elements. A simple sum of scores from all 7 movement elements may not be the optimal predictor of stroke volume. For that, we used least-squares regression, which appropriately weighted each movement score to best predict stroke size. We applied this method to individual movements’ scores provided by the expert (yellow points) and separately to movement element scores predicted by the network (blue points). To prevent model overfitting, we used leave-one-rat-out cross-validation as described in the Methods. Results in this figure also show that when the network is trained to reproduce scores of individual movement elements, its stroke volume predictions are more similar to human scoring (compare to Fig 2C). This suggests that the network in Fig 2C learned to use temporal combinations of movement features to predict stroke severity. When predicting severity from individual movement elements, the absence of information about temporal relations reduces predictability. (C) Correlation coefficient between individual movement scores (calculated from expert scores) shows that movement components closer in time tend to have higher correlation of impairment scores. We found that out of 7 movement elements, the lift component had the highest correlation with stroke volume (RLift = 0.3592; RAim = 0.2879; RAdv = 0.2446; RPron = 0.2651; RGrasp = 0.2889; RSup1 = −0.0 [file pbio.3000516.s005.tif]

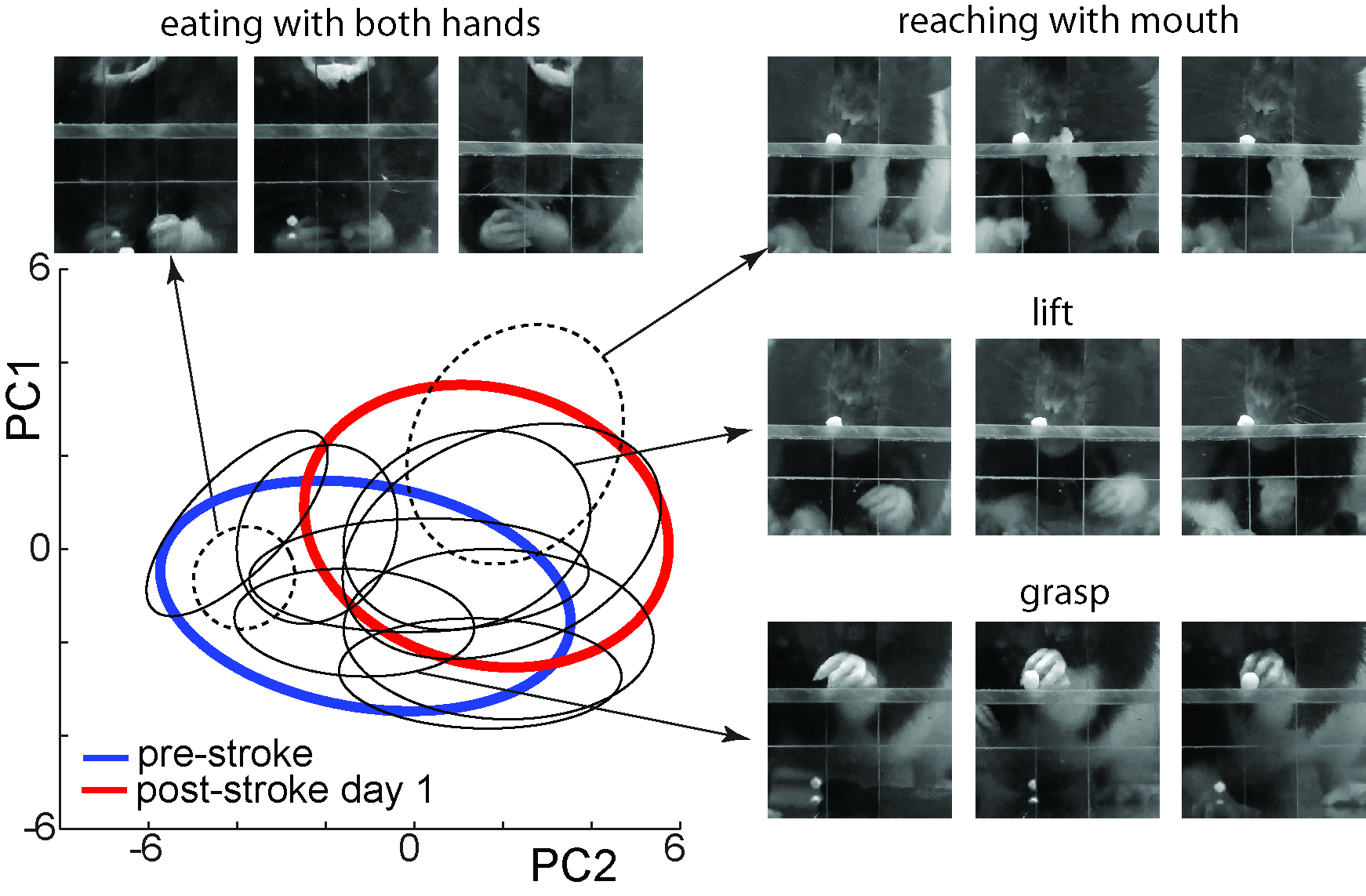

Supplement: S6 Fig — We selected frames from the closest to the subcluster center. Each of three frames shown for a single subcluster is selected from a different rat. Note that frames at the bottom show examples of successful and unsuccessful grasps. Rationale for dividing data in 40 subclusters: The typical number of movement elements defined by experts in reaching task is between 7 and 10. Each movement element can significantly differ between stroke and control animals; for example, the pronation cluster may be divided in two or more distinct subclusters corresponding to level of impairment (Fig 5). Moreover, at the beginning and at the end of a trial, there could be other movements—e.g., rearing or walking—which likely would form separate clusters in feature space. Therefore, to be able to differentiate all those possibly distinct movement subclusters, we decided to divide the data into 40 clusters. The fit of an ellipse to a given set of points was done by minimizing the least-squares criterion. Ellipses were only used for data visualization and were not used for data analyses. (TIF) [file pbio.3000516.s006.tif]

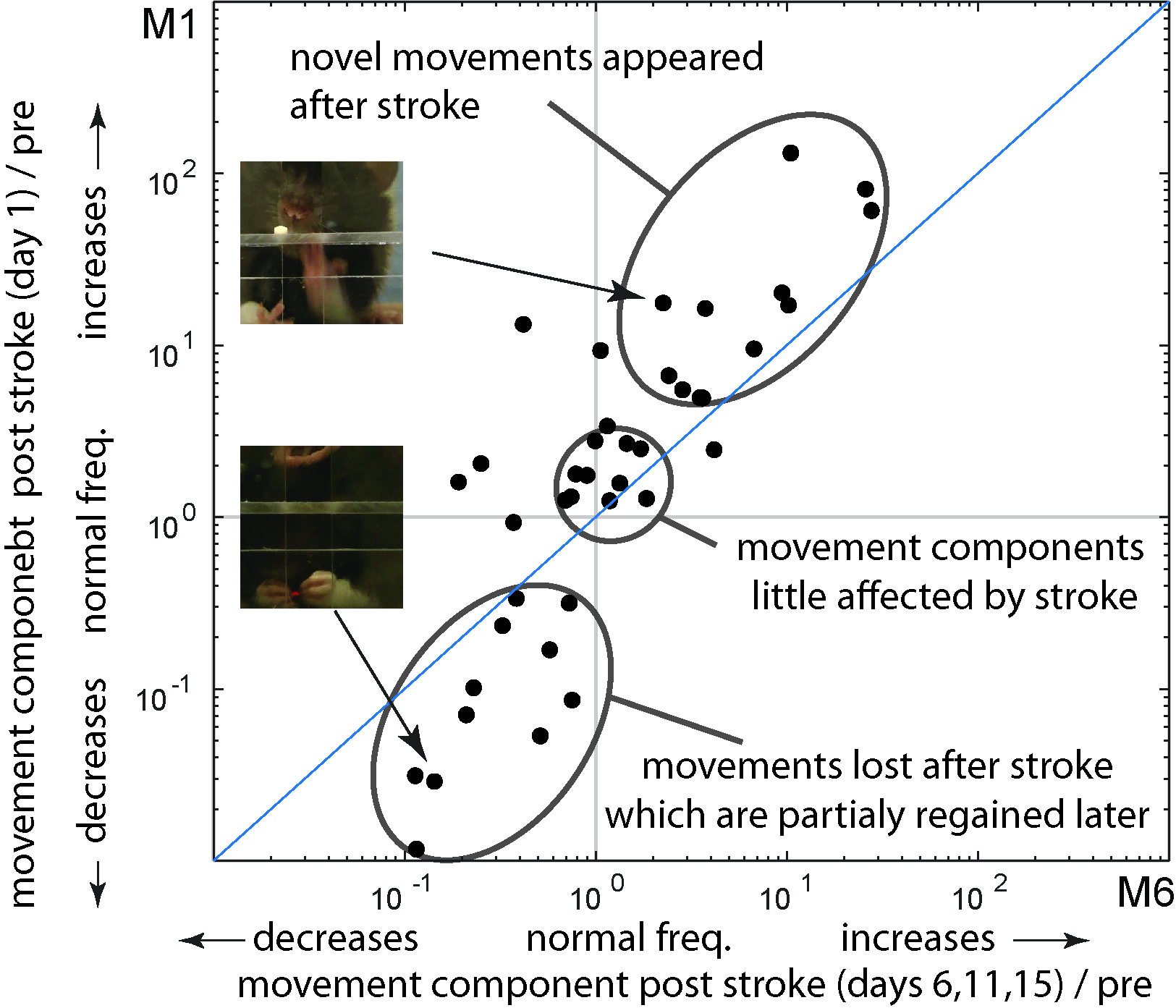

Supplement: S7 Fig — Each dot represents a subcluster corresponding to single movement component. y-Axis (M1) is a ratio of movement probability on day 1 after stroke and prestroke, and x-axis (M6) is a ratio of movement probabilities on days 6–15 after stroke in relation to prestroke probability. Specifically, here we investigated changes during the poststroke period across all subclusters (movement components). For this, within each subcluster j, we counted the number of points from the control period (p0j), the number of points from day 1 after stroke (p1j), and the sum of points from days 6, 11, and 15 after stroke (p6j) (those counts were then converted to probabilities by dividing by the total number of points on a given day). To see the changes in relation to the prestroke period for each subcluster, we calculated measures: M1j = p1j/p0j and M6j = p6j/p0j (for numerical stability to avoid division by 0, we added a small epsilon = 0.0001 to all pj). In other words, M1j is a ratio of red bar (day 1) to blue bar (day before stroke) in Fig 6Ba or 6Bb. Plotting M1 versus M6 showed that about the third of the movement components effectively disappeared after stroke, with a similar number of new movement elements emerging after stroke. The distributions of points above the diagonal for “novel” movements and below the diagonal for “lost” movements indicates improvements in movement during the recovery period (days 6–15). To estimate how “elongated” the distribution of points along diagonal is, we calculated the correlation coefficient between log(M1) and log(M6) values for each rat separately. We found that more elongated distributions correlated with higher values of movement disability scores (r = 0.58, p < 0.001). Those analyses illustrate that the internal network representation can express complex movements in a simple low-dimensional representation, which allows for a detailed tracking of stroke recovery. (TIF) [file pbio.3000516.s007.tif]

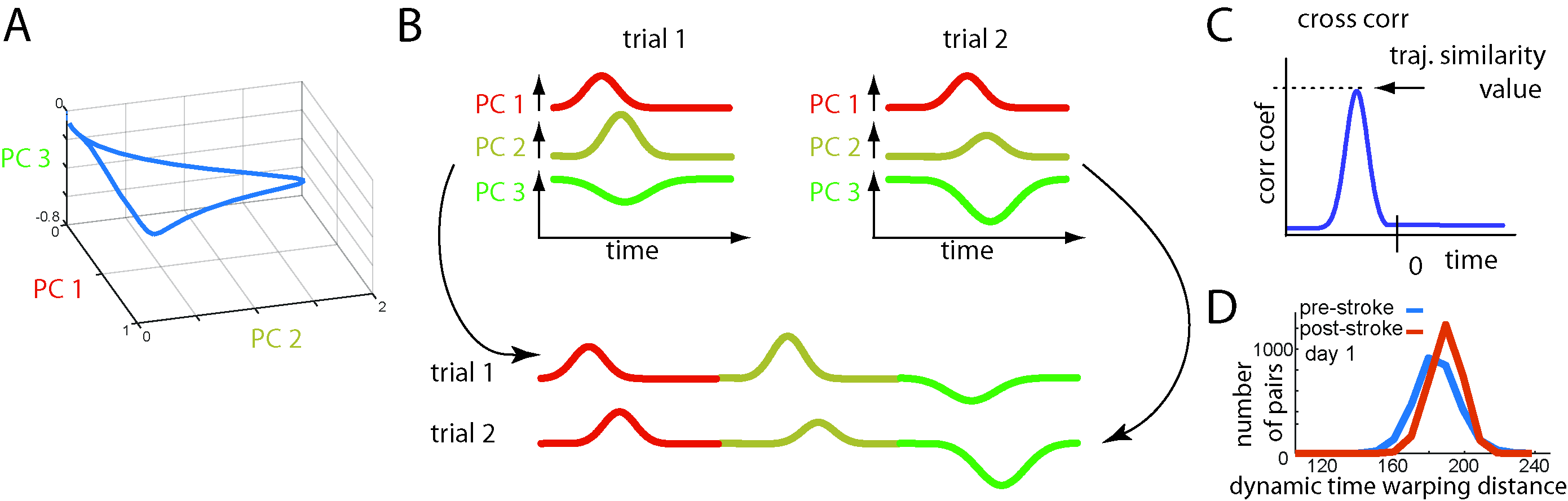

Supplement: S8 Fig — (A) Any point in a movement trajectory is defined by values in PCs’ coordinates. To compare two trajectories, we concatenated the values of PC components in a single vector, separately for each trial. (B) Next, we calculated the cross-correlogram between both vectors, and the maximum value of the cross-correlogram was used as a measure of similarity between two movement trajectories. (C) Using the cross-correlogram enables the detection of similar movements, even if one movement started at a different time in relation to the beginning of the video clip. We used the first 7 PCs to measure the similarity of trajectories. We have chosen 7 PCs based on examination of eigenvalues, but changing the number of PCs between 5 and 15 did not affect our conclusions. (D) To measure distance between trajectories in a manner robust to changes in movement velocity, we also applied dynamic time warping (https://www.mathworks.com/matlabcentral/fileexchange/43156-dynamic-time-warping-dtw). Using dynamic time warping gave similar results to that using cross-correlation measure (compare with Fig 7; note that in this plot, smaller values of distance indicate larger similarity of trajectories). PC, principal component. (TIF) [file pbio.3000516.s008.tif]

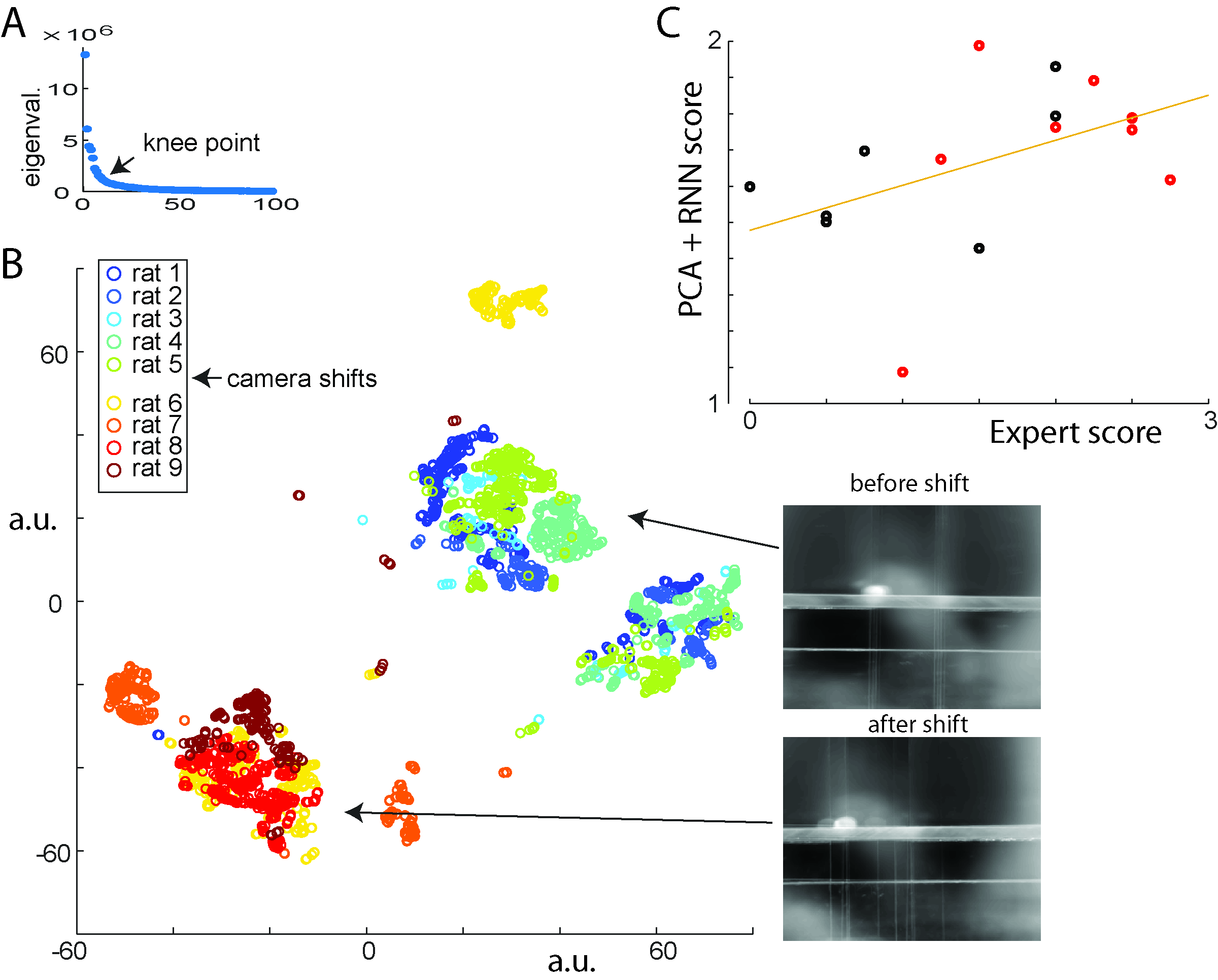

Supplement: S9 Fig — (A) As a control experiment, we applied PCA to all video frames to use it as features. As a result, each video frame was represented by 20 principal components, accounting for most of the variance. Next, we applied a least-squares regression to predict the expert scores from PCA scores. This did not give a significant result (r = −0.1 p = 0.69). For selecting the number of PCA components, we use the scree plot method [74]. Specifically, in the above scree plot of eigenvalues, we selected a “knee” point at which values level off. This is a generally used rule of thumb, as there is no theoretically optimal method for selecting the number of PCA components. To ensure this does not introduce bias, we varied the number of selected PCA components between 10 and 100, which gave consistent results. (B) To investigate why our predictions from PCA scores failed, we visualized the first 20 principal components in 2D using t-SNE [42], a nonlinear dimensionality reduction technique. Each point corresponds to a single frame in the t-SNE projection. Data from each rat are marked with a different color. For clarity, only data from nine rats and 1 day are shown, when the camera was accidentally moved after filming rat #5. The camera shift caused large change in PCA features as evident by separate cluster for rats 6–9. The average frame for rats 1–5 and rats 6–9 is shown on the right side. (C) Using 2,048 principal components and the RNN improved the predictions toward expert scores. However, it was still markedly worse than using 2,048 ConvNet features and RNN (compare to Fig 2B). This is likely due to fact that convolutional networks with pooling layers, as used in our approach, can extract features which are robust to spatial shifts [75]. We also asked whether the dimensionality of the featurized image data changes before and after stroke. To test this, we did SVD on image features from ConvNet separately for data before and after stroke. We could not detect significant difference [file pbio.3000516.s009.tif]

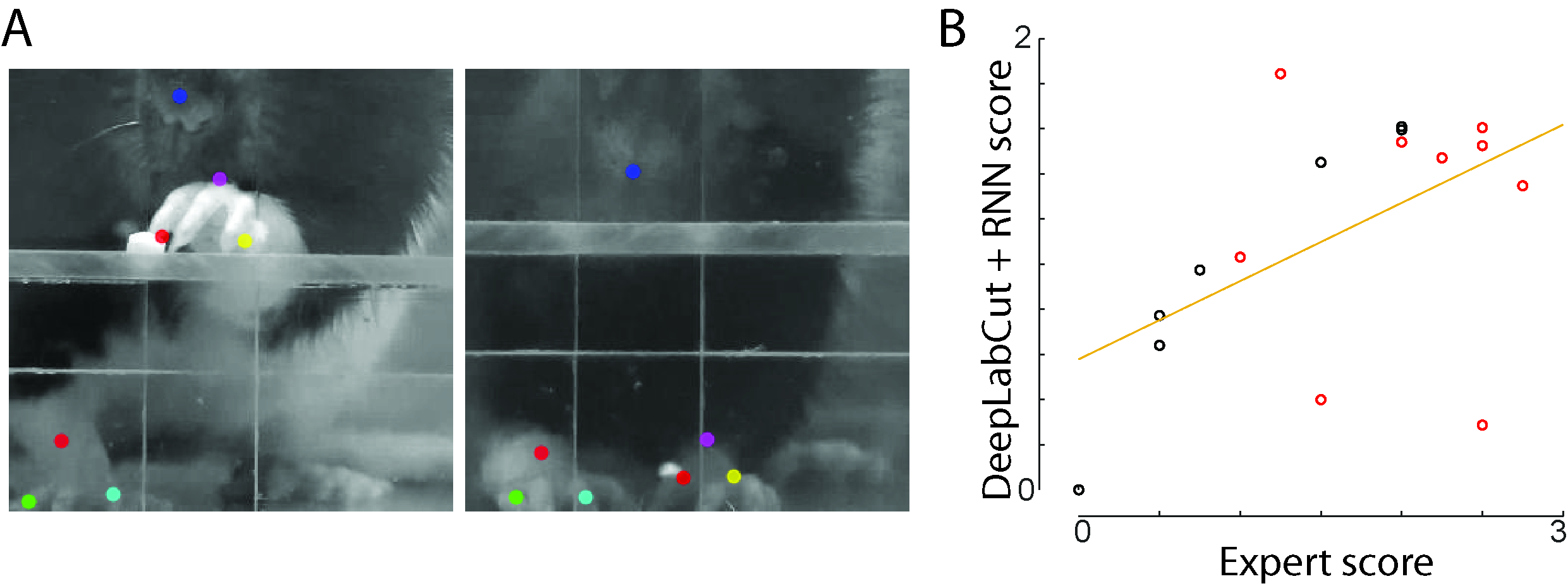

Supplement: S10 Fig — (A) Sample frames with marked body parts by DeepLabCut. (B) We used the RNN to predict expert scores from coordinates of points marked by DeepLabCut and from its confidence levels. The correlation coefficient between predicted and actual expert scores was r = 0.53, p = 0.036. All x- and y-coordinates were divided by 300 to be in the 0–1 range. For points assigned to body parts that were not visible in a frame or were difficult to identify, DeepLabCut gave a confidence level close to 0. Adding 4 more markers on body parts did not significantly change predictions. We also tried other software to track body parts: LEAP [43], which gave comparable results to DeepLabCut. RNN, recurrent neural network. (TIF) [file pbio.3000516.s010.tif]

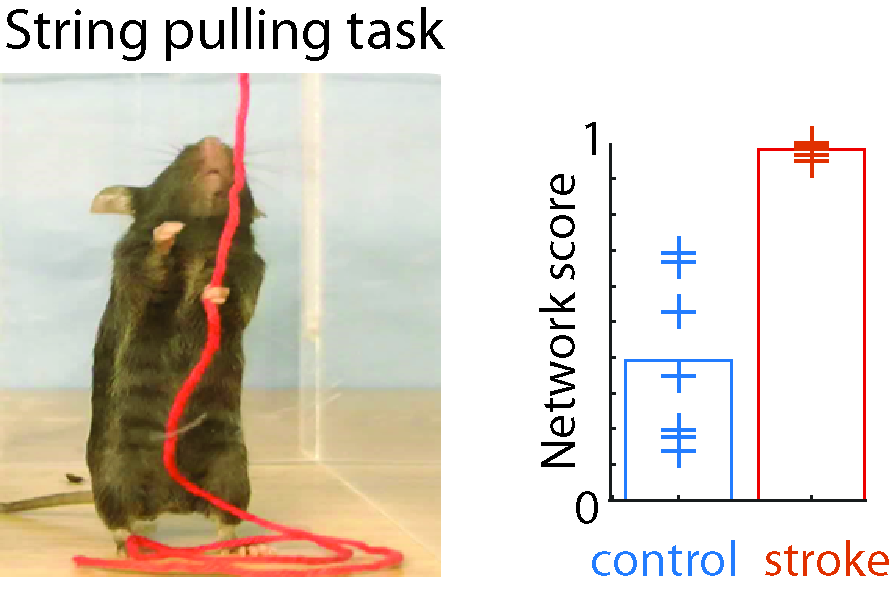

Supplement: S11 Fig — (Left) Sample frame of a mouse during the string-pulling task. For this task, we trained the network to discriminate control from stroke mice. (Right) Network scores of movement deficits. Bars show group average, and crosses show scores for individual mice. The network learned to discriminate between both groups with 100% accuracy (note: no overlap in scores between groups). String-pulling task: This task examines the coordination of bilateral hand and arm movements used in spontaneous string pulling. Procedures for training mice in the string-pulling task were based on an earlier description of the behavior [5]. Briefly, 11 Chat-CreAi32 mice (5 females, 6 males) that were 3–5 months old, weighed 20–30 g, and were raised at the Canadian Centre for Behavioural Neuroscience Vivarium at the University of Lethbridge were weighed and placed on food restriction 3 days prior to training. Food reinforcement on the end of the string was used as motivation for string pulling. Mice were weighed daily to maintain body weight at 90% of pre-restriction weight. They were given additional food in their home cage 2 hours after completion of daily training/testing. On each training/testing day, animals were individually placed in a clear plastic container for transport to the testing room. Mice were trained for 3 days to pull a 90-cm-long piece of string hanging from the top of a transparent Plexiglas box to obtain the food reward tied at the end, and then they were filmed. They underwent photothrombotic stroke induction in their primary forelimb somatosensory area and were filmed in the string-pulling task before stroke and on day 4 poststroke. Animals were kept on food restriction throughout the experiment. No scoring system has been developed for this task; thus, we only trained network to classify stroke versus control condition with pre- and poststroke videos. (TIF) [file pbio.3000516.s011.tif]
