# Supplementary material for: Correction: Correction: Correction: Data-driven analyses of motor impairments in animal models of neurological disorders
Source: PLoS Biol. 2026 Nov 5;24(11):e3004090. doi: 10.1371/journal.pbio.3004090 (PMC13739121; doi:10.1371/journal.pbio.3004090)
Supplement: Supplementary file 2 [file pbio.3004090.s002.pdf]

CORRECTION

Correction: Correction: Data-driven analyses of motor impairments in animal models of neurological disorders

The *PLOS One* Staff

There is an error in the citation for author Behroo Mirza Agha in the correction published on February 8, 2024. The correct citation is: Ryait H, Bermudez-Contreras E, Harvey M, Faraji J, Mirza Agha B, Gomez-Palacio Schjetnan A, and colleagues (2019) Data-driven analyses of motor impairments in animal models of neurological disorders. PLOS Biology 17(11): e3000516. https://doi.org/10.1371/journal.pbio.3000516.

References

1. Ryait H, Bermudez-Contreras E, Harvey M, Faraji J, Mirza Agha B, Gomez-Palacio Schjetnan A, et al. Data-driven analyses of motor impairments in animal models of neurological disorders. PLoS Biol. 2019;17(11):e3000516. https://doi.org/10.1371/journal.pbio.3000516 PMID: 31751328
2. Ryait H, Bermudez-Contreras E, Harvey M, Faraji J, Agha BM, Schjetnan AG-P, et al. Correction: Data-driven analyses of motor impairments in animal models of neurological disorders. PLoS Biol. 2024;22(2):e3002521. https://doi.org/10.1371/journal.pbio.3002521 PMID: 38330553

OPEN ACCESS

Citation: The *PLOS One* Staff (2026) Correction: Correction: Data-driven analyses of motor impairments in animal models of neurological disorders. PLoS Biol 24(9): e3004030. https://doi.org/10.1371/journal.pbio.3004030

Published: September 29, 2026

Copyright: © 2026 The *PLOS One* Staff. This is an open access article distributed under the terms of the Creative Commons Attribution License, which permits unrestricted use, distribution, and reproduction in any medium, provided the original author and source are credited.
